# Supplementary material for: Mental health and mental health help-seeking behaviors among first-generation voluntary African migrants: A systematic review
Source: PLoS One. 2024 Mar 18;19(3):e0298634. doi: 10.1371/journal.pone.0298634 (PMC10947684; doi:10.1371/journal.pone.0298634)
Supplement: S1 File — (DOCX) [file pone.0298634.s001.docx]

**Search Strategy**

(MM "Mental Health") OR "Mental health" OR (MH "Mental Disorders+") OR "mental wellbeing" OR "mental health symptoms" OR "mental health problems" OR (MH "Mental Health Recovery") OR "mental health issues" OR "prevalence of mental health problems" OR "mental health risk" OR (MM "Depression") OR (MH "Depressive Disorder, Major") OR (MH "Depressive Disorder") OR (MH "Depression, Postpartum") OR (MM "Bipolar Disorder") OR (MH "Seasonal Affective Disorder") OR (MH "Mood Disorders") OR (MH "Affective Disorders, Psychotic") OR (MH "Affective Symptoms") OR (MH "Bipolar and Related Disorders") OR (MM "Anxiety+") OR (MM "Anxiety Disorders+") OR (MM "Phobia, Social") OR (MH "Anxiety, Separation") OR (MM "Stress, Psychological+") OR (MH "Stress Disorders, Post-Traumatic") OR (MH "Stress Disorders, Traumatic") OR (MH "Stress Disorders, Traumatic, Acute") OR (MH "Sleep Disorders, Intrinsic") OR "PTSD" OR (MH "Sleep Wake Disorders") OR (MH "Sleep Disorders, Circadian Rhythm") OR (MH "Sleep") OR (MH "Sleep Duration") OR (MH "Sleep Quality") OR (MH "Fatigue") OR (MH "Mental Fatigue") OR (MH "Hospitals, Chronic Disease") OR (MH "Psychological Distress") OR "psychiatric problems" OR "psychotic disorders"

AND

(MM "Mental Health Help-Seeking Behavior”) (MM “Help-Seeking Behavior") (MM "Help-Seeking Behavior") (MM "Help-Seeking Behavior") OR (MM "Social Behavior Disorders+") OR (MH "Drug-Seeking Behavior") OR (MH "Exploratory Behavior") OR "help-seeking support" OR (MM "Host-Seeking Behavior") OR "help seeking support" OR (MH "Helping Behavior") OR "mental help-seeking attitude" OR "mental health assistance" OR "coping strategies" OR "coping" OR "coping mechanisms" OR "coping style" OR "mental health literacy" OR "mental health education" OR (MH "Mental Health Services") OR "mental health information" OR (MH "Health Literacy")

AND

[

Migrant Or Migrants OR immigrant* OR "African migrant" OR (MH "West African People") OR (MH "Central African People") OR (MH "North African People") OR (MH "African People") OR (MH "Southern African People") OR (MH "Black or African American") OR (MH "East African People") OR (MH "Sub-Saharan African People") OR "Africans in diaspora" OR "African migrants" OR (MH "Africa South of the Sahara") OR (MH "Emigrants and Immigrants") OR (MH "Transients and Migrants") OR (MH "Emigration and Immigration") OR (MH "Undocumented Immigrants") OR "foreigners" OR "people of colour" OR "minority population" OR "first generation migrants" OR "first generation immigrants" OR "voluntary migrants" OR "voluntary African migrants"

OR

Algeria OR Angola OR Benin OR Botswana OR "Burkina Faso" OR Burundi OR "Cabo Verde" OR Cameroon OR "Central African Republic" OR Chad OR Comoros OR "Republic of the Congo" OR "Republic of the Cote d’Ivoire" OR Djibouti OR Egypt OR "Equatorial Guinea" OR Eritrea OR Eswatini OR Ethiopia OR Gabon OR Gambia OR Ghana OR "Guinea OR Guinea-Bissau" OR Kenya OR "Lesotho OR Liberia" OR Libya OR Madagascar OR Malawi OR Mali OR Mauritania OR Mauritius OR Morocco OR Mozambique OR Namibia OR Niger OR Nigeria OR Rwanda OR "Sao Tome and Principe" OR Senegal OR Seychelles OR "Sierra Leone" OR Somalia OR "South Africa" OR "South Sudan" OR Sudan OR Tanzania OR Togo OR Tunisia OR Uganda OR Zambia OR Zimbabwe

]

AND

(MM "Adult+") OR "man" OR (MH "Men") OR (MH "Women") OR "woman" OR "individuals" OR (MH "Persons") OR "youth" OR (MH "Young Adult")
