# Supplementary material for: Mental health and mental health help-seeking behaviors among first-generation voluntary African migrants: A systematic review
Source: PLoS One. 2024 Mar 18;19(3):e0298634. doi: 10.1371/journal.pone.0298634 (PMC10947684; doi:10.1371/journal.pone.0298634)
Supplement: S1 Appendix — A. CINAHL Search Strategy 15.07.2022. B. Embase Search Strategy 15.07.2022. C. Medline Complete Search Strategy 15.07.2022. D. PsychInfo Search Strategy 15.07.2022. (ZIP) [file pone.0298634.s003.zip › S1B_Appendix.txt]

☐	#61	**'coping'**:ab,ti	83,191
☐	#60	**'coping strategies'**:ab,ti	20,641
☐	#59	#47 OR #48 OR #49 OR #50 OR #51 OR #52 OR #53 OR #54 OR #55 OR #56 OR #57 OR #58	10,935
☐	#58	**'support-seeking'**:ab,ti	734
☐	#57	**'mental health assistance'**:ab,ti	109
☐	#56	**'mental help-seeking** :ab,ti	
