# Supplementary material for: Mental health and mental health help-seeking behaviors among first-generation voluntary African migrants: A systematic review
Source: PLoS One. 2024 Mar 18;19(3):e0298634. doi: 10.1371/journal.pone.0298634 (PMC10947684; doi:10.1371/journal.pone.0298634)
Supplement: S1 Appendix — A. CINAHL Search Strategy 15.07.2022. B. Embase Search Strategy 15.07.2022. C. Medline Complete Search Strategy 15.07.2022. D. PsychInfo Search Strategy 15.07.2022. (ZIP) [file pone.0298634.s003.zip › S1D_Appendix.txt]

Friday, July 15, 2022 8:50:57 AM

#	Query	Limiters/Expanders	Last Run Via	Results
S112	(S47 AND S73 AND S82 AND S108)	Limiters - Published Date: 20120101-20221231; English<br>Search modes - Boolean/Phrase	Interface - EBSCOhost Research Databases<br>Search Screen - Advanced Search<br>Database - APA PsycInfo	3,404
S111	(S47 AND S73 AND S82 AND S108)	Limiters - English<br>Search modes - Boolean/Phrase	Interface - EBSCOhost Research Databases<br>Search Screen - Advanced Search<br>Database - APA PsycInfo	5,967
S110	(S47 AND S73 AND S82 AND S108)	Search modes - Boolean/Phrase	Interface - EBSCOhost Research Databases<br>Search Screen - Advanced Search<br>Database - APA PsycInfo	6,209
S109	(S47 AND S73 AND S82 AND S98 AND S108)	Search modes - Boolean/Phrase	Interface - EBSCOhost Research Databases<br>Search Screen - Advanced Search<br>Database - APA PsycInfo	595
S108	(S99 OR S100 OR S101 OR S102 OR S103 OR S104 OR S105 OR S106 OR S107)	Search modes - Boolean/Phrase	Interface - EBSCOhost Research Databases<br>Search Screen - Advanced Search<br>Database - APA PsycInfo	1,531,183
S107	TI young adults OR AB young adults	Search modes - Boolean/Phrase	Interface - EBSCOhost Research Databases<br>Search Screen - Advanced Search<br>Database - APA PsycInfo	57,811
S106	TI youth OR AB youth	Search modes - Boolean/Phrase	Interface - EBSCOhost Research Databases<br>Search Screen - Advanced Search<br>Database - APA PsycInfo	110,539
S105	TI individuals OR AB individuals	Search modes - Boolean/Phrase	Interface - EBSCOhost Research Databases	749,383

			Search Screen - Advanced Search<br>Database - APA PsycInfo	
S104	TI women OR AB woman	Search modes - Boolean/Phrase	Interface - EBSCOhost Research Databases<br>Search Screen - Advanced Search<br>Database - APA PsycInfo	334,837
S103	TI women OR AB women	Search modes - Boolean/Phrase	Interface - EBSCOhost Research Databases<br>Search Screen - Advanced Search<br>Database - APA PsycInfo	334,837
S102	TI men OR AB men	Search modes - Boolean/Phrase	Interface - EBSCOhost Research Databases<br>Search Screen - Advanced Search<br>Database - APA PsycInfo	231,443
S101	TI man OR AB man	Search modes - Boolean/Phrase	Interface - EBSCOhost Research Databases<br>Search Screen - Advanced Search<br>Database - APA PsycInfo	231,443
S100	TI adults OR AB adults	Search modes - Boolean/Phrase	Interface - EBSCOhost Research Databases<br>Search Screen - Advanced Search<br>Database - APA PsycInfo	456,948
S99	TI Adolescen OR AB Adolescen	Search modes - Boolean/Phrase	Interface - EBSCOhost Research Databases<br>Search Screen - Advanced Search<br>Database - APA PsycInfo	24
S98	S83 OR S84 OR S85 OR S86 OR S87 OR S88 OR S89 OR S90 OR S91 OR S92 OR S93 OR S94 OR S95 OR S96 OR S97	Search modes - Boolean/Phrase	Interface - EBSCOhost Research Databases<br>Search Screen - Advanced Search<br>Database - APA PsycInfo	131,396
S97	TI first generation immigrants OR AB first generation immigrants	Search modes - Boolean/Phrase	Interface - EBSCOhost Research Databases<br>Search Screen - Advanced Search<br>Database - APA PsycInfo	950

S96	TI first generation migrants OR AB first generation migrants	Search modes - Boolean/Phrase	Interface - EBSCOhost Research Databases<br>Search Screen - Advanced Search<br>Database - APA PsycInfo	172
S95	TI minority population* OR AB minority population*	Search modes - Boolean/Phrase	Interface - EBSCOhost Research Databases<br>Search Screen - Advanced Search<br>Database - APA PsycInfo	4,904
S94	TI people of colour OR AB people of colour	Search modes - Boolean/Phrase	Interface - EBSCOhost Research Databases<br>Search Screen - Advanced Search<br>Database - APA PsycInfo	2,971
S93	TI foreigners OR AB foreigners	Search modes - Boolean/Phrase	Interface - EBSCOhost Research Databases<br>Search Screen - Advanced Search<br>Database - APA PsycInfo	1,251
S92	TI undocumented migrants OR AB undocumented immigrants	Search modes - Boolean/Phrase	Interface - EBSCOhost Research Databases<br>Search Screen - Advanced Search<br>Database - APA PsycInfo	796
S91	TI undocumented migrants OR AB undocumented migrants	Search modes - Boolean/Phrase	Interface - EBSCOhost Research Databases<br>Search Screen - Advanced Search<br>Database - APA PsycInfo	215
S90	TI immigrants OR AB immigrants	Search modes - Boolean/Phrase	Interface - EBSCOhost Research Databases<br>Search Screen - Advanced Search<br>Database - APA PsycInfo	29,001
S89	TI immigration OR AB immigration	Search modes - Boolean/Phrase	Interface - EBSCOhost Research Databases<br>Search Screen - Advanced Search<br>Database - APA PsycInfo	11,218
S88	TI migration OR AB migration	Search modes - Boolean/Phrase	Interface - EBSCOhost Research Databases	19,880

			Search Screen - Advanced Search<br>Database - APA PsycInfo	
S87	TI migrants OR AB migrants	Search modes - Boolean/Phrase	Interface - EBSCOhost Research Databases<br>Search Screen - Advanced Search<br>Database - APA PsycInfo	11,689
S86	TI sub-Saharan African* OR AB sub-Saharan African*	Search modes - Boolean/Phrase	Interface - EBSCOhost Research Databases<br>Search Screen - Advanced Search<br>Database - APA PsycInfo	939
S85	TI Africans in diaspora OR AB Africans in diaspora	Search modes - Boolean/Phrase	Interface - EBSCOhost Research Databases<br>Search Screen - Advanced Search<br>Database - APA PsycInfo	287
S84	TI Africans OR AB Africans	Search modes - Boolean/Phrase	Interface - EBSCOhost Research Databases<br>Search Screen - Advanced Search<br>Database - APA PsycInfo	70,650
S83	TI African migrants OR AB African migrants	Search modes - Boolean/Phrase	Interface - EBSCOhost Research Databases<br>Search Screen - Advanced Search<br>Database - APA PsycInfo	270
S82	S74 OR S75 OR S76 OR S77 OR S78 OR S79 OR S80 OR S81	Search modes - Boolean/Phrase	Interface - EBSCOhost Research Databases<br>Search Screen - Advanced Search<br>Database - APA PsycInfo	1,450,883
S81	TI eyewitness OR AB eyewitness	Search modes - Boolean/Phrase	Interface - EBSCOhost Research Databases<br>Search Screen - Advanced Search<br>Database - APA PsycInfo	3,245
S80	TI witness OR AB witness	Search modes - Boolean/Phrase	Interface - EBSCOhost Research Databases<br>Search Screen - Advanced Search<br>Database - APA PsycInfo	11,356

S79	TI experience* OR AB experience*	Search modes - Boolean/Phrase	Interface - EBSCOhost Research Databases<br>Search Screen - Advanced Search<br>Database - APA PsycInfo	712,981
S78	TI perspective* OR AB perspective*	Search modes - Boolean/Phrase	Interface - EBSCOhost Research Databases<br>Search Screen - Advanced Search<br>Database - APA PsycInfo	303,736
S77	TI lived experience* OR AB lived experience*	Search modes - Boolean/Phrase	Interface - EBSCOhost Research Databases<br>Search Screen - Advanced Search<br>Database - APA PsycInfo	20,000
S76	TI account OR AB account	Search modes - Boolean/Phrase	Interface - EBSCOhost Research Databases<br>Search Screen - Advanced Search<br>Database - APA PsycInfo	187,818
S75	TI rates OR AB rates	Search modes - Boolean/Phrase	Interface - EBSCOhost Research Databases<br>Search Screen - Advanced Search<br>Database - APA PsycInfo	343,580
S74	TI Prevalence OR AB Prevalence	Search modes - Boolean/Phrase	Interface - EBSCOhost Research Databases<br>Search Screen - Advanced Search<br>Database - APA PsycInfo	129,393
S73	(S61 OR S67 OR S72)	Search modes - Boolean/Phrase	Interface - EBSCOhost Research Databases<br>Search Screen - Advanced Search<br>Database - APA PsycInfo	118,912
S72	(S68 OR S69 OR S70 OR S71)	Search modes - Boolean/Phrase	Interface - EBSCOhost Research Databases<br>Search Screen - Advanced Search<br>Database - APA PsycInfo	16,180
S71	TI health literacy OR AB health literacy	Search modes - Boolean/Phrase	Interface - EBSCOhost Research Databases	5,060

			Search Screen - Advanced Search<br>Database - APA PsycInfo	
S70	TI mental health infor* OR AB mental health infor*	Search modes - Boolean/Phrase	Interface - EBSCOhost Research Databases<br>Search Screen - Advanced Search<br>Database - APA PsycInfo	4,914
S69	TI mental health educa* OR AB mental health educa*	Search modes - Boolean/Phrase	Interface - EBSCOhost Research Databases<br>Search Screen - Advanced Search<br>Database - APA PsycInfo	6,746
S68	TI mental health literacy scale OR AB mental health literacy	Search modes - Boolean/Phrase	Interface - EBSCOhost Research Databases<br>Search Screen - Advanced Search<br>Database - APA PsycInfo	1,158
S67	S62 OR S63 OR S64 OR S65 OR S66	Search modes - Boolean/Phrase	Interface - EBSCOhost Research Databases<br>Search Screen - Advanced Search<br>Database - APA PsycInfo	84,237
S66	TI coping tools OR AB coping tools	Search modes - Boolean/Phrase	Interface - EBSCOhost Research Databases<br>Search Screen - Advanced Search<br>Database - APA PsycInfo	342
S65	TI coping style OR AB coping style	Search modes - Boolean/Phrase	Interface - EBSCOhost Research Databases<br>Search Screen - Advanced Search<br>Database - APA PsycInfo	8,257
S64	TI coping mechanisms OR AB coping mechanisms	Search modes - Boolean/Phrase	Interface - EBSCOhost Research Databases<br>Search Screen - Advanced Search<br>Database - APA PsycInfo	5,013
S63	TI coping OR AB coping	Search modes - Boolean/Phrase	Interface - EBSCOhost Research Databases<br>Search Screen - Advanced Search<br>Database - APA PsycInfo	84,237

S62	TI coping strategies OR AB coping strategies	Search modes - Boolean/Phrase	Interface - EBSCOhost Research Databases<br>Search Screen - Advanced Search<br>Database - APA PsycInfo	24,315
S61	(S48 OR S49 OR S50 OR S51 OR S52 OR S53 OR S54 OR S55 OR S56 OR S57 OR S58 OR S59 OR S60)	Search modes - Boolean/Phrase	Interface - EBSCOhost Research Databases<br>Search Screen - Advanced Search<br>Database - APA PsycInfo	22,407
S60	TI support-seeking OR AB support-seeking	Search modes - Boolean/Phrase	Interface - EBSCOhost Research Databases<br>Search Screen - Advanced Search<br>Database - APA PsycInfo	1,069
S59	TI mental health assistance OR AB mental health assistance	Search modes - Boolean/Phrase	Interface - EBSCOhost Research Databases<br>Search Screen - Advanced Search<br>Database - APA PsycInfo	601
S58	TI mental help-seeking attitude* OR AB mental help-seeking attitude*	Search modes - Boolean/Phrase	Interface - EBSCOhost Research Databases<br>Search Screen - Advanced Search<br>Database - APA PsycInfo	190
S57	TI mental health support OR AB mental health support	Search modes - Boolean/Phrase	Interface - EBSCOhost Research Databases<br>Search Screen - Advanced Search<br>Database - APA PsycInfo	8,046
S56	TI helping behavior OR AB helping behavior	Search modes - Boolean/Phrase	Interface - EBSCOhost Research Databases<br>Search Screen - Advanced Search<br>Database - APA PsycInfo	3,442
S55	TI help seeking support OR AB help seeking support	Search modes - Boolean/Phrase	Interface - EBSCOhost Research Databases<br>Search Screen - Advanced Search<br>Database - APA PsycInfo	619
S54	TI help-seeking support OR AB help-seeking	Search modes - Boolean/Phrase	Interface - EBSCOhost Research Databases	463

	support		Search Screen - Advanced Search<br>Database - APA PsycInfo	
S53	TI help seeking behavior OR AB help seeking behavior	Search modes - Boolean/Phrase	Interface - EBSCOhost Research Databases<br>Search Screen - Advanced Search<br>Database - APA PsycInfo	3,390
S52	TI help seeking behaviour OR AB help seeking behaviour	Search modes - Boolean/Phrase	Interface - EBSCOhost Research Databases<br>Search Screen - Advanced Search<br>Database - APA PsycInfo	3,390
S51	TI help-seeking behavior OR AB help-seeking behavior	Search modes - Boolean/Phrase	Interface - EBSCOhost Research Databases<br>Search Screen - Advanced Search<br>Database - APA PsycInfo	3,259
S50	TI help seeking behaviour OR AB help-seeking behaviour	Search modes - Boolean/Phrase	Interface - EBSCOhost Research Databases<br>Search Screen - Advanced Search<br>Database - APA PsycInfo	3,274
S49	TI help seeking behaviour OR AB help-seeking behaviour	Search modes - Boolean/Phrase	Interface - EBSCOhost Research Databases<br>Search Screen - Advanced Search<br>Database - APA PsycInfo	3,274
S48	TI help-seeking OR AB help-seeking	Search modes - Boolean/Phrase	Interface - EBSCOhost Research Databases<br>Search Screen - Advanced Search<br>Database - APA PsycInfo	9,476
S47	S26 OR S33 OR S39 OR S46	Search modes - Boolean/Phrase	Interface - EBSCOhost Research Databases<br>Search Screen - Advanced Search<br>Database - APA PsycInfo	432,305
S46	S40 OR S41 OR S42 OR S43 OR S44 OR S45	Search modes - Boolean/Phrase	Interface - EBSCOhost Research Databases<br>Search Screen - Advanced Search<br>Database - APA PsycInfo	378,932

S45	TI psychotic disorders OR AB psychotic disorders	Search modes - Boolean/Phrase	Interface - EBSCOhost Research Databases<br>Search Screen - Advanced Search<br>Database - APA PsycInfo	13,697
S44	TI psychiatric problems OR AB psychiatric problems	Search modes - Boolean/Phrase	Interface - EBSCOhost Research Databases<br>Search Screen - Advanced Search<br>Database - APA PsycInfo	8,008
S43	TI psychological distress OR AB psychological distress	Search modes - Boolean/Phrase	Interface - EBSCOhost Research Databases<br>Search Screen - Advanced Search<br>Database - APA PsycInfo	23,105
S42	TI psychological effects OR AB psychological*	Search modes - Boolean/Phrase	Interface - EBSCOhost Research Databases<br>Search Screen - Advanced Search<br>Database - APA PsycInfo	358,101
S41	TI psychological problems OR AB psychological impact	Search modes - Boolean/Phrase	Interface - EBSCOhost Research Databases<br>Search Screen - Advanced Search<br>Database - APA PsycInfo	10,666
S40	TI psychological problems OR AB psychological problems	Search modes - Boolean/Phrase	Interface - EBSCOhost Research Databases<br>Search Screen - Advanced Search<br>Database - APA PsycInfo	15,548
S39	(S34 OR S35 OR S36 OR S37 OR S38)	Search modes - Boolean/Phrase	Interface - EBSCOhost Research Databases<br>Search Screen - Advanced Search<br>Database - APA PsycInfo	28,636
S38	TI mental fatigue OR AB mental fatigue	Search modes - Boolean/Phrase	Interface - EBSCOhost Research Databases<br>Search Screen - Advanced Search<br>Database - APA PsycInfo	1,565
S37	TI tiredness OR AB tiredness	Search modes - Boolean/Phrase	Interface - EBSCOhost Research Databases	1,501

			Search Screen - Advanced Search<br>Database - APA PsycInfo	
S36	TI chronic fatigue syndrome OR AB chronic fatigue syndrome	Search modes - Boolean/Phrase	Interface - EBSCOhost Research Databases<br>Search Screen - Advanced Search<br>Database - APA PsycInfo	2,458
S35	TI chronic fatigue OR AB chronic fatigue	Search modes - Boolean/Phrase	Interface - EBSCOhost Research Databases<br>Search Screen - Advanced Search<br>Database - APA PsycInfo	3,348
S34	TI fatigue OR AB fatigue	Search modes - Boolean/Phrase	Interface - EBSCOhost Research Databases<br>Search Screen - Advanced Search<br>Database - APA PsycInfo	27,470
S33	S27 OR S28 OR S29 OR S30 OR S31 OR S32	Search modes - Boolean/Phrase	Interface - EBSCOhost Research Databases<br>Search Screen - Advanced Search<br>Database - APA PsycInfo	35,385
S32	TI daytime sleepiness` OR AB daytime sleepiness	Search modes - Boolean/Phrase	Interface - EBSCOhost Research Databases<br>Search Screen - Advanced Search<br>Database - APA PsycInfo	3,406
S31	TI sleeplessness OR AB sleeplessness	Search modes - Boolean/Phrase	Interface - EBSCOhost Research Databases<br>Search Screen - Advanced Search<br>Database - APA PsycInfo	433
S30	TI insomnia OR AB insomnia	Search modes - Boolean/Phrase	Interface - EBSCOhost Research Databases<br>Search Screen - Advanced Search<br>Database - APA PsycInfo	13,736
S29	TI sleep problems OR AB sleep problems	Search modes - Boolean/Phrase	Interface - EBSCOhost Research Databases<br>Search Screen - Advanced Search<br>Database - APA PsycInfo	6,819

S28	TI sleep disturbance* OR AB sleep disturbance*	Search modes - Boolean/Phrase	Interface - EBSCOhost Research Databases<br>Search Screen - Advanced Search<br>Database - APA PsycInfo	10,575
S27	TI sleep disorders OR AB sleep disorders	Search modes - Boolean/Phrase	Interface - EBSCOhost Research Databases<br>Search Screen - Advanced Search<br>Database - APA PsycInfo	11,693
S26	((TI PTSD OR AB PTSD) AND (S1 AND S2 AND S3 AND S4 AND S5 AND S6 AND S7 AND S8 AND S9 AND S10 AND S11 AND S12 AND S13 AND S14 AND S15 AND S16 AND S17 AND S18 AND S19 AND S20 AND S21 AND S22 AND S23 AND S24 AND S25)) AND (S1 OR S2 OR S3 OR S4 OR S5 OR S6 OR S7 OR S8 OR S9 OR S10 OR S11 OR S12 OR S13 OR S14 OR S15 OR S16 OR S17 OR S18 OR S19 OR S20 OR S21 OR S22 OR S23 OR S24 OR S25)	Search modes - Boolean/Phrase	Interface - EBSCOhost Research Databases<br>Search Screen - Advanced Search<br>Database - APA PsycInfo	238
S25	TI PTSD OR AB PTSD	Search modes - Boolean/Phrase	Interface - EBSCOhost Research Databases<br>Search Screen - Advanced Search<br>Database - APA PsycInfo	36,787
S24	TI post-traumatic stress disorder* OR AB post-traumatic stress disorder*	Search modes - Boolean/Phrase	Interface - EBSCOhost Research Databases<br>Search Screen - Advanced Search<br>Database - APA PsycInfo	12,181
S23	TI stress disorder* OR AB stress disorder*	Search modes - Boolean/Phrase	Interface - EBSCOhost Research Databases<br>Search Screen - Advanced	46,419

			Search<br>Database - APA PsycInfo	
S22	TI physiological stress* OR AB physiological stress*	Search modes - Boolean/Phrase	Interface - EBSCOhost Research Databases<br>Search Screen - Advanced Search<br>Database - APA PsycInfo	5,637
S21	TI psychological stress* OR AB psychological stress*	Search modes - Boolean/Phrase	Interface - EBSCOhost Research Databases<br>Search Screen - Advanced Search<br>Database - APA PsycInfo	15,282
S20	TI stress OR AB stress	Search modes - Boolean/Phrase	Interface - EBSCOhost Research Databases<br>Search Screen - Advanced Search<br>Database - APA PsycInfo	242,637
S19	TI generalized anxiety disorder OR AB generalized anxiety disorder	Search modes - Boolean/Phrase	Interface - EBSCOhost Research Databases<br>Search Screen - Advanced Search<br>Database - APA PsycInfo	8,298
S18	TI anxiety disorder OR AB anxiety disorder	Search modes - Boolean/Phrase	Interface - EBSCOhost Research Databases<br>Search Screen - Advanced Search<br>Database - APA PsycInfo	50,359
S17	TI anxiety OR AB anxiety	Search modes - Boolean/Phrase	Interface - EBSCOhost Research Databases<br>Search Screen - Advanced Search<br>Database - APA PsycInfo	219,197
S16	TI bipolar disorder OR AB bipolar disorder	Search modes - Boolean/Phrase	Interface - EBSCOhost Research Databases<br>Search Screen - Advanced Search<br>Database - APA PsycInfo	33,650
S15	TI seasonal affective disorder OR AB seasonal affective disorder	Search modes - Boolean/Phrase	Interface - EBSCOhost Research Databases<br>Search Screen - Advanced Search<br>Database - APA PsycInfo	1,242

S14	TI depressive disorder OR AB depressive disorder	Search modes - Boolean/Phrase	Interface - EBSCOhost Research Databases<br>Search Screen - Advanced Search<br>Database - APA PsycInfo	40,867
S13	TI depression OR AB depression	Search modes - Boolean/Phrase	Interface - EBSCOhost Research Databases<br>Search Screen - Advanced Search<br>Database - APA PsycInfo	269,627
S12	TI mental wellbeing OR AB mental wellbeing	Search modes - Boolean/Phrase	Interface - EBSCOhost Research Databases<br>Search Screen - Advanced Search<br>Database - APA PsycInfo	2,298
S11	TI mental disorders OR AB mental disorders	Search modes - Boolean/Phrase	Interface - EBSCOhost Research Databases<br>Search Screen - Advanced Search<br>Database - APA PsycInfo	68,675
S10	TI mental health risk OR AB mental health risk	Search modes - Boolean/Phrase	Interface - EBSCOhost Research Databases<br>Search Screen - Advanced Search<br>Database - APA PsycInfo	8,448
S9	TI prevalence of mental health problems OR AB prevalence of mental health problems	Search modes - Boolean/Phrase	Interface - EBSCOhost Research Databases<br>Search Screen - Advanced Search<br>Database - APA PsycInfo	935
S8	TI mental health issues OR AB mental health issues	Search modes - Boolean/Phrase	Interface - EBSCOhost Research Databases<br>Search Screen - Advanced Search<br>Database - APA PsycInfo	10,804
S7	TI mental health problems OR AB mental health problems	Search modes - Boolean/Phrase	Interface - EBSCOhost Research Databases<br>Search Screen - Advanced Search<br>Database - APA PsycInfo	23,220
S6	TI mental health symptoms OR AB mental health symptoms	Search modes - Boolean/Phrase	Interface - EBSCOhost Research Databases<br>Search Screen - Advanced	6,301

			Search<br>Database - APA PsycInfo	
S5	TI mental health status OR AB mental health status	Search modes - Boolean/Phrase	Interface - EBSCOhost Research Databases<br>Search Screen - Advanced Search<br>Database - APA PsycInfo	5,586
S4	TI mental wellbeing OR AB mental wellbeing	Search modes - Boolean/Phrase	Interface - EBSCOhost Research Databases<br>Search Screen - Advanced Search<br>Database - APA PsycInfo	2,298
S3	TI mental health disorder* OR AB mental health disorder*	Search modes - Boolean/Phrase	Interface - EBSCOhost Research Databases<br>Search Screen - Advanced Search<br>Database - APA PsycInfo	11,673
S2	TI mental distress OR AB mental distress	Search modes - Boolean/Phrase	Interface - EBSCOhost Research Databases<br>Search Screen - Advanced Search<br>Database - APA PsycInfo	4,180
S1	TI mental health OR AB mental health	Search modes - Boolean/Phrase	Interface - EBSCOhost Research Databases<br>Search Screen - Advanced Search<br>Database - APA PsycInfo	215,920
