# Supplementary material for: Mental health and mental health help-seeking behaviors among first-generation voluntary African migrants: A systematic review
Source: PLoS One. 2024 Mar 18;19(3):e0298634. doi: 10.1371/journal.pone.0298634 (PMC10947684; doi:10.1371/journal.pone.0298634)
Supplement: S1 Appendix — A. CINAHL Search Strategy 15.07.2022. B. Embase Search Strategy 15.07.2022. C. Medline Complete Search Strategy 15.07.2022. D. PsychInfo Search Strategy 15.07.2022. (ZIP) [file pone.0298634.s003.zip › S1A_Appendix.txt]

MY
EBSCOhost

Friday, July 15, 2022 9:27:22 AM

| # | Query | Limiters/Expanders | Last Run Via | Results |
|---|---|---|---|---|
| S113 | (S47 AND S73 AND S82 AND S108) | Search modes - Boolean/Phrase | Interface - EBSCOhost Research Databases<br>Search Screen - Advanced Search<br>Database - CINAHL Complete | 2,802 |
| S112 | (S47 AND S73 AND S82 AND S108) | Limiters - Published Date: 20120101-20221231; English Language<br>Search modes - Boolean/Phrase | Interface - EBSCOhost Research Databases<br>Search Screen - Advanced Search<br>Database - CINAHL Complete | 1,874 |
| S111 | (S47 AND S73 AND S82 AND S108) | Limiters - English Language<br>Search modes - Boolean/Phrase | Interface - EBSCOhost Research Databases<br>Search Screen - Advanced Search<br>Database - CINAHL Complete | 2,743 |
| S110 | (S47 AND S73 AND S82 AND S108) | Search modes - Boolean/Phrase | Interface - EBSCOhost Research Databases<br>Search Screen - Advanced Search<br>Database - CINAHL Complete | 2,802 |
| S109 | (S47 AND S73 AND S82 AND S98 AND S108) | Search modes - Boolean/Phrase | Interface - EBSCOhost Research Databases<br>Search Screen - Advanced Search<br>Database - CINAHL Complete | 149 |
| S108 | (S99 OR S100 OR S101 OR S102 OR S103 OR S104 OR S105 OR S106 OR S107) | Search modes - Boolean/Phrase | Interface - EBSCOhost Research Databases<br>Search Screen - Advanced Search<br>Database - CINAHL Complete | 1,338,307 |
| S107 | TI young adults OR AB young adults | Search modes - Boolean/Phrase | Interface - EBSCOhost Research Databases<br>Search Screen - Advanced Search<br>Database - CINAHL Complete | 43,942 |
| S106 | TI youth OR AB youth | Search modes - | Interface - EBSCOhost | 58,594 |

| | | | | |
|---|---|---|---|---|
| | | Boolean/Phrase | Research Databases<br>Search Screen - Advanced Search<br>Database - CINAHL Complete | |
| S105 | TI individuals OR AB individuals | Search modes - Boolean/Phrase | Interface - EBSCOhost Research Databases<br>Search Screen - Advanced Search<br>Database - CINAHL Complete | 440,441 |
| S104 | TI women OR AB woman | Search modes - Boolean/Phrase | Interface - EBSCOhost Research Databases<br>Search Screen - Advanced Search<br>Database - CINAHL Complete | 480,825 |
| S103 | TI women OR AB women | Search modes - Boolean/Phrase | Interface - EBSCOhost Research Databases<br>Search Screen - Advanced Search<br>Database - CINAHL Complete | 480,825 |
| S102 | TI men OR AB men | Search modes - Boolean/Phrase | Interface - EBSCOhost Research Databases<br>Search Screen - Advanced Search<br>Database - CINAHL Complete | 233,242 |
| S101 | TI man OR AB man | Search modes - Boolean/Phrase | Interface - EBSCOhost Research Databases<br>Search Screen - Advanced Search<br>Database - CINAHL Complete | 233,242 |
| S100 | TI adults OR AB adults | Search modes - Boolean/Phrase | Interface - EBSCOhost Research Databases<br>Search Screen - Advanced Search<br>Database - CINAHL Complete | 415,919 |
| S99 | TI Adolescen OR AB Adolescen | Search modes - Boolean/Phrase | Interface - EBSCOhost Research Databases<br>Search Screen - Advanced Search<br>Database - CINAHL Complete | 18 |
| S98 | S83 OR S84 OR S85 OR S86 OR S87 OR S88 OR S89 OR S90 OR S91 OR | Search modes - Boolean/Phrase | Interface - EBSCOhost Research Databases<br>Search Screen - Advanced | 106,328 |

| | | | | |
|---|---|---|---|---|
| | S92 OR S93 OR S94 OR S95 OR S96 OR S97 | | Search<br>Database - CINAHL Complete | |
| S97 | TI first generation immigrants OR AB first generation immigrants | Search modes - Boolean/Phrase | Interface - EBSCOhost Research Databases<br>Search Screen - Advanced Search<br>Database - CINAHL Complete | 400 |
| S96 | TI first generation migrants OR AB first generation migrants | Search modes - Boolean/Phrase | Interface - EBSCOhost Research Databases<br>Search Screen - Advanced Search<br>Database - CINAHL Complete | 131 |
| S95 | TI minority population* OR AB minority population* | Search modes - Boolean/Phrase | Interface - EBSCOhost Research Databases<br>Search Screen - Advanced Search<br>Database - CINAHL Complete | 3,884 |
| S94 | TI people of colour OR AB people of colour | Search modes - Boolean/Phrase | Interface - EBSCOhost Research Databases<br>Search Screen - Advanced Search<br>Database - CINAHL Complete | 1,046 |
| S93 | TI foreigners OR AB foreigners | Search modes - Boolean/Phrase | Interface - EBSCOhost Research Databases<br>Search Screen - Advanced Search<br>Database - CINAHL Complete | 333 |
| S92 | TI undocumented migrants OR AB undocumented immigrants | Search modes - Boolean/Phrase | Interface - EBSCOhost Research Databases<br>Search Screen - Advanced Search<br>Database - CINAHL Complete | 523 |
| S91 | TI undocumented migrants OR AB undocumented migrants | Search modes - Boolean/Phrase | Interface - EBSCOhost Research Databases<br>Search Screen - Advanced Search<br>Database - CINAHL Complete | 228 |
| S90 | TI immigrants OR AB immigrants | Search modes - Boolean/Phrase | Interface - EBSCOhost Research Databases<br>Search Screen - Advanced Search<br>Database - CINAHL Complete | 14,993 |

| | | | | |
|---|---|---|---|---|
| S89 | TI immigration OR AB immigration | Search modes - Boolean/Phrase | Interface - EBSCOhost Research Databases<br>Search Screen - Advanced Search<br>Database - CINAHL Complete | 4,859 |
| S88 | TI migration OR AB migration | Search modes - Boolean/Phrase | Interface - EBSCOhost Research Databases<br>Search Screen - Advanced Search<br>Database - CINAHL Complete | 27,122 |
| S87 | TI migrants OR AB migrants | Search modes - Boolean/Phrase | Interface - EBSCOhost Research Databases<br>Search Screen - Advanced Search<br>Database - CINAHL Complete | 8,498 |
| S86 | TI sub-Saharan African* OR AB sub-Saharan African* | Search modes - Boolean/Phrase | Interface - EBSCOhost Research Databases<br>Search Screen - Advanced Search<br>Database - CINAHL Complete | 1,730 |
| S85 | TI Africans in diaspora OR AB Africans in diaspora | Search modes - Boolean/Phrase | Interface - EBSCOhost Research Databases<br>Search Screen - Advanced Search<br>Database - CINAHL Complete | 78 |
| S84 | TI Africans OR AB Africans | Search modes - Boolean/Phrase | Interface - EBSCOhost Research Databases<br>Search Screen - Advanced Search<br>Database - CINAHL Complete | 54,813 |
| S83 | TI African migrants OR AB African migrants | Search modes - Boolean/Phrase | Interface - EBSCOhost Research Databases<br>Search Screen - Advanced Search<br>Database - CINAHL Complete | 270 |
| S82 | S74 OR S75 OR S76 OR S77 OR S78 OR S79 OR S80 OR S81 | Search modes - Boolean/Phrase | Interface - EBSCOhost Research Databases<br>Search Screen - Advanced Search<br>Database - CINAHL Complete | 1,350,061 |
| S81 | TI eyewitness OR AB eyewitness | Search modes - Boolean/Phrase | Interface - EBSCOhost Research Databases<br>Search Screen - Advanced | 344 |

| | | | | |
|---|---|---|---|---|
| | | | Search<br>Database - CINAHL Complete | |
| S80 | TI witness OR AB witness | Search modes - Boolean/Phrase | Interface - EBSCOhost Research Databases<br>Search Screen - Advanced Search<br>Database - CINAHL Complete | 4,057 |
| S79 | TI experience* OR AB experience* | Search modes - Boolean/Phrase | Interface - EBSCOhost Research Databases<br>Search Screen - Advanced Search<br>Database - CINAHL Complete | 493,680 |
| S78 | TI perspective* OR AB perspective* | Search modes - Boolean/Phrase | Interface - EBSCOhost Research Databases<br>Search Screen - Advanced Search<br>Database - CINAHL Complete | 160,018 |
| S77 | TI lived experience* OR AB lived experience* | Search modes - Boolean/Phrase | Interface - EBSCOhost Research Databases<br>Search Screen - Advanced Search<br>Database - CINAHL Complete | 10,427 |
| S76 | TI account OR AB account | Search modes - Boolean/Phrase | Interface - EBSCOhost Research Databases<br>Search Screen - Advanced Search<br>Database - CINAHL Complete | 87,058 |
| S75 | TI rates OR AB rates | Search modes - Boolean/Phrase | Interface - EBSCOhost Research Databases<br>Search Screen - Advanced Search<br>Database - CINAHL Complete | 593,540 |
| S74 | TI Prevalence OR AB Prevalence | Search modes - Boolean/Phrase | Interface - EBSCOhost Research Databases<br>Search Screen - Advanced Search<br>Database - CINAHL Complete | 217,906 |
| S73 | (S61 OR S67 OR S72) | Search modes - Boolean/Phrase | Interface - EBSCOhost Research Databases<br>Search Screen - Advanced Search<br>Database - CINAHL Complete | 67,413 |

| | | | | |
|---|---|---|---|---|
| S72 | (S68 OR S69 OR S70 OR S71) | Search modes - Boolean/Phrase | Interface - EBSCOhost Research Databases<br>Search Screen - Advanced Search<br>Database - CINAHL Complete | 15,411 |
| S71 | TI health literacy OR AB health literacy | Search modes - Boolean/Phrase | Interface - EBSCOhost Research Databases<br>Search Screen - Advanced Search<br>Database - CINAHL Complete | 9,318 |
| S70 | TI mental health infor* OR AB mental health infor* | Search modes - Boolean/Phrase | Interface - EBSCOhost Research Databases<br>Search Screen - Advanced Search<br>Database - CINAHL Complete | 2,906 |
| S69 | TI mental health educa* OR AB mental health educa* | Search modes - Boolean/Phrase | Interface - EBSCOhost Research Databases<br>Search Screen - Advanced Search<br>Database - CINAHL Complete | 3,508 |
| S68 | TI mental health literacy scale OR AB mental health literacy | Search modes - Boolean/Phrase | Interface - EBSCOhost Research Databases<br>Search Screen - Advanced Search<br>Database - CINAHL Complete | 699 |
| S67 | S62 OR S63 OR S64 OR S65 OR S66 | Search modes - Boolean/Phrase | Interface - EBSCOhost Research Databases<br>Search Screen - Advanced Search<br>Database - CINAHL Complete | 42,173 |
| S66 | TI coping tools OR AB coping tools | Search modes - Boolean/Phrase | Interface - EBSCOhost Research Databases<br>Search Screen - Advanced Search<br>Database - CINAHL Complete | 209 |
| S65 | TI coping style OR AB coping style | Search modes - Boolean/Phrase | Interface - EBSCOhost Research Databases<br>Search Screen - Advanced Search<br>Database - CINAHL Complete | 2,822 |
| S64 | TI coping mechanisms OR AB coping mechanisms | Search modes - Boolean/Phrase | Interface - EBSCOhost Research Databases<br>Search Screen - Advanced | 2,274 |

| | | | | |
|---|---|---|---|---|
| | | | Search<br>Database - CINAHL Complete | |
| S63 | TI coping OR AB coping | Search modes - Boolean/Phrase | Interface - EBSCOhost Research Databases<br>Search Screen - Advanced Search<br>Database - CINAHL Complete | 42,173 |
| S62 | TI coping strategies OR AB coping strategies | Search modes - Boolean/Phrase | Interface - EBSCOhost Research Databases<br>Search Screen - Advanced Search<br>Database - CINAHL Complete | 12,362 |
| S61 | (S48 OR S49 OR S50 OR S51 OR S52 OR S53 OR S54 OR S55 OR S56 OR S57 OR S58 OR S59 OR S60) | Search modes - Boolean/Phrase | Interface - EBSCOhost Research Databases<br>Search Screen - Advanced Search<br>Database - CINAHL Complete | 11,825 |
| S60 | TI support-seeking OR AB support-seeking | Search modes - Boolean/Phrase | Interface - EBSCOhost Research Databases<br>Search Screen - Advanced Search<br>Database - CINAHL Complete | 421 |
| S59 | TI mental health assistance OR AB mental health assistance | Search modes - Boolean/Phrase | Interface - EBSCOhost Research Databases<br>Search Screen - Advanced Search<br>Database - CINAHL Complete | 324 |
| S58 | TI mental help-seeking attitude* OR AB mental help-seeking attitude* | Search modes - Boolean/Phrase | Interface - EBSCOhost Research Databases<br>Search Screen - Advanced Search<br>Database - CINAHL Complete | 64 |
| S57 | TI mental health support OR AB mental health support | Search modes - Boolean/Phrase | Interface - EBSCOhost Research Databases<br>Search Screen - Advanced Search<br>Database - CINAHL Complete | 5,891 |
| S56 | TI helping behavior OR AB helping behavior | Search modes - Boolean/Phrase | Interface - EBSCOhost Research Databases<br>Search Screen - Advanced Search<br>Database - CINAHL Complete | 671 |

| | | | | |
|---|---|---|---|---|
| S55 | TI help seeking support OR AB help seeking support | Search modes - Boolean/Phrase | Interface - EBSCOhost Research Databases<br>Search Screen - Advanced Search<br>Database - CINAHL Complete | 282 |
| S54 | TI help-seeking support OR AB help-seeking support | Search modes - Boolean/Phrase | Interface - EBSCOhost Research Databases<br>Search Screen - Advanced Search<br>Database - CINAHL Complete | 199 |
| S53 | TI help seeking behavior OR AB help seeking behavior | Search modes - Boolean/Phrase | Interface - EBSCOhost Research Databases<br>Search Screen - Advanced Search<br>Database - CINAHL Complete | 1,673 |
| S52 | TI help seeking behaviour OR AB help seeking behaviour | Search modes - Boolean/Phrase | Interface - EBSCOhost Research Databases<br>Search Screen - Advanced Search<br>Database - CINAHL Complete | 1,673 |
| S51 | TI help-seeking behavior OR AB help-seeking behavior | Search modes - Boolean/Phrase | Interface - EBSCOhost Research Databases<br>Search Screen - Advanced Search<br>Database - CINAHL Complete | 1,592 |
| S50 | TI help seeking behaviour OR AB help-seeking behaviour | Search modes - Boolean/Phrase | Interface - EBSCOhost Research Databases<br>Search Screen - Advanced Search<br>Database - CINAHL Complete | 1,601 |
| S49 | TI help seeking behaviour OR AB help-seeking behaviour | Search modes - Boolean/Phrase | Interface - EBSCOhost Research Databases<br>Search Screen - Advanced Search<br>Database - CINAHL Complete | 1,601 |
| S48 | TI help-seeking OR AB help-seeking | Search modes - Boolean/Phrase | Interface - EBSCOhost Research Databases<br>Search Screen - Advanced Search<br>Database - CINAHL Complete | 4,603 |
| S47 | S26 OR S33 OR S39 OR S46 | Search modes - Boolean/Phrase | Interface - EBSCOhost Research Databases<br>Search Screen - Advanced | 180,483 |

| | | | | |
|---|---|---|---|---|
| | | | Search<br>Database - CINAHL Complete | |
| S46 | S40 OR S41 OR S42 OR S43 OR S44 OR S45 | Search modes - Boolean/Phrase | Interface - EBSCOhost Research Databases<br>Search Screen - Advanced Search<br>Database - CINAHL Complete | 114,996 |
| S45 | TI psychotic disorders OR AB psychotic disorders | Search modes - Boolean/Phrase | Interface - EBSCOhost Research Databases<br>Search Screen - Advanced Search<br>Database - CINAHL Complete | 4,627 |
| S44 | TI psychiatric problems OR AB psychiatric problems | Search modes - Boolean/Phrase | Interface - EBSCOhost Research Databases<br>Search Screen - Advanced Search<br>Database - CINAHL Complete | 2,621 |
| S43 | TI psychological distress OR AB psychological distress | Search modes - Boolean/Phrase | Interface - EBSCOhost Research Databases<br>Search Screen - Advanced Search<br>Database - CINAHL Complete | 14,368 |
| S42 | TI psychological effects OR AB psychological* | Search modes - Boolean/Phrase | Interface - EBSCOhost Research Databases<br>Search Screen - Advanced Search<br>Database - CINAHL Complete | 107,137 |
| S41 | TI psychological problems OR AB psychological impact | Search modes - Boolean/Phrase | Interface - EBSCOhost Research Databases<br>Search Screen - Advanced Search<br>Database - CINAHL Complete | 5,467 |
| S40 | TI psychological problems OR AB psychological problems | Search modes - Boolean/Phrase | Interface - EBSCOhost Research Databases<br>Search Screen - Advanced Search<br>Database - CINAHL Complete | 5,058 |
| S39 | (S34 OR S35 OR S36 OR S37 OR S38) | Search modes - Boolean/Phrase | Interface - EBSCOhost Research Databases<br>Search Screen - Advanced Search<br>Database - CINAHL Complete | 45,111 |

| | | | | |
|---|---|---|---|---|
| S38 | TI mental fatigue OR AB mental fatigue | Search modes - Boolean/Phrase | Interface - EBSCOhost Research Databases<br>Search Screen - Advanced Search<br>Database - CINAHL Complete | 1,102 |
| S37 | TI tiredness OR AB tiredness | Search modes - Boolean/Phrase | Interface - EBSCOhost Research Databases<br>Search Screen - Advanced Search<br>Database - CINAHL Complete | 1,733 |
| S36 | TI chronic fatigue syndrome OR AB chronic fatigue syndrome | Search modes - Boolean/Phrase | Interface - EBSCOhost Research Databases<br>Search Screen - Advanced Search<br>Database - CINAHL Complete | 2,685 |
| S35 | TI chronic fatigue OR AB chronic fatigue | Search modes - Boolean/Phrase | Interface - EBSCOhost Research Databases<br>Search Screen - Advanced Search<br>Database - CINAHL Complete | 4,275 |
| S34 | TI fatigue OR AB fatigue | Search modes - Boolean/Phrase | Interface - EBSCOhost Research Databases<br>Search Screen - Advanced Search<br>Database - CINAHL Complete | 43,827 |
| S33 | S27 OR S28 OR S29 OR S30 OR S31 OR S32 | Search modes - Boolean/Phrase | Interface - EBSCOhost Research Databases<br>Search Screen - Advanced Search<br>Database - CINAHL Complete | 29,127 |
| S32 | TI daytime sleepiness` OR AB daytime sleepiness | Search modes - Boolean/Phrase | Interface - EBSCOhost Research Databases<br>Search Screen - Advanced Search<br>Database - CINAHL Complete | 2,812 |
| S31 | TI sleeplessness OR AB sleeplessness | Search modes - Boolean/Phrase | Interface - EBSCOhost Research Databases<br>Search Screen - Advanced Search<br>Database - CINAHL Complete | 308 |
| S30 | TI insomnia OR AB insomnia | Search modes - Boolean/Phrase | Interface - EBSCOhost Research Databases<br>Search Screen - Advanced | 10,497 |

| | | | | |
|---|---|---|---|---|
| | | | Search<br>Database - CINAHL Complete | |
| S29 | TI sleep problems OR AB sleep problems | Search modes - Boolean/Phrase | Interface - EBSCOhost Research Databases<br>Search Screen - Advanced Search<br>Database - CINAHL Complete | 5,287 |
| S28 | TI sleep disturbance* OR AB sleep disturbance* | Search modes - Boolean/Phrase | Interface - EBSCOhost Research Databases<br>Search Screen - Advanced Search<br>Database - CINAHL Complete | 8,482 |
| S27 | TI sleep disorders OR AB sleep disorders | Search modes - Boolean/Phrase | Interface - EBSCOhost Research Databases<br>Search Screen - Advanced Search<br>Database - CINAHL Complete | 8,775 |
| S26 | ((TI PTSD OR AB PTSD) AND (S1 AND S2 AND S3 AND S4 AND S5 AND S6 AND S7 AND S8 AND S9 AND S10 AND S11 AND S12 AND S13 AND S14 AND S15 AND S16 AND S17 AND S18 AND S19 AND S20 AND S21 AND S22 AND S23 AND S24 AND S25)) AND (S1 OR S2 OR S3 OR S4 OR S5 OR S6 OR S7 OR S8 OR S9 OR S10 OR S11 OR S12 OR S13 OR S14 OR S15 OR S16 OR S17 OR S18 OR S19 OR S20 OR S21 OR S22 OR S23 OR S24 OR S25) | Search modes - Boolean/Phrase | Interface - EBSCOhost Research Databases<br>Search Screen - Advanced Search<br>Database - CINAHL Complete | 1,895 |
| S25 | TI PTSD OR AB PTSD | Search modes - Boolean/Phrase | Interface - EBSCOhost Research Databases<br>Search Screen - Advanced Search<br>Database - CINAHL Complete | 13,420 |
| S24 | TI post-traumatic stress disorder* OR AB post- | Search modes - Boolean/Phrase | Interface - EBSCOhost Research Databases | 6,468 |

| | | | | |
|---|---|---|---|---|
| | traumatic stress disorder* | | Search Screen - Advanced Search<br>Database - CINAHL Complete | |
| S23 | TI stress disorder* OR AB stress disorder* | Search modes - Boolean/Phrase | Interface - EBSCOhost Research Databases<br>Search Screen - Advanced Search<br>Database - CINAHL Complete | 18,791 |
| S22 | TI physiological stress* OR AB physiological stress* | Search modes - Boolean/Phrase | Interface - EBSCOhost Research Databases<br>Search Screen - Advanced Search<br>Database - CINAHL Complete | 2,170 |
| S21 | TI psychological stress* OR AB psychological stress* | Search modes - Boolean/Phrase | Interface - EBSCOhost Research Databases<br>Search Screen - Advanced Search<br>Database - CINAHL Complete | 6,800 |
| S20 | TI stress OR AB stress | Search modes - Boolean/Phrase | Interface - EBSCOhost Research Databases<br>Search Screen - Advanced Search<br>Database - CINAHL Complete | 167,368 |
| S19 | TI generalized anxiety disorder OR AB generalized anxiety disorder | Search modes - Boolean/Phrase | Interface - EBSCOhost Research Databases<br>Search Screen - Advanced Search<br>Database - CINAHL Complete | 2,920 |
| S18 | TI anxiety disorder OR AB anxiety disorder | Search modes - Boolean/Phrase | Interface - EBSCOhost Research Databases<br>Search Screen - Advanced Search<br>Database - CINAHL Complete | 18,039 |
| S17 | TI anxiety OR AB anxiety | Search modes - Boolean/Phrase | Interface - EBSCOhost Research Databases<br>Search Screen - Advanced Search<br>Database - CINAHL Complete | 97,448 |
| S16 | TI bipolar disorder OR AB bipolar disorder | Search modes - Boolean/Phrase | Interface - EBSCOhost Research Databases<br>Search Screen - Advanced Search<br>Database - CINAHL Complete | 11,868 |

| | | | | |
|---|---|---|---|---|
| S15 | TI seasonal affective disorder OR AB seasonal affective disorder | Search modes - Boolean/Phrase | Interface - EBSCOhost Research Databases<br>Search Screen - Advanced Search<br>Database - CINAHL Complete | 383 |
| S14 | TI depressive disorder OR AB depressive disorder | Search modes - Boolean/Phrase | Interface - EBSCOhost Research Databases<br>Search Screen - Advanced Search<br>Database - CINAHL Complete | 14,660 |
| S13 | TI depression OR AB depression | Search modes - Boolean/Phrase | Interface - EBSCOhost Research Databases<br>Search Screen - Advanced Search<br>Database - CINAHL Complete | 143,664 |
| S12 | TI mental wellbeing OR AB mental wellbeing | Search modes - Boolean/Phrase | Interface - EBSCOhost Research Databases<br>Search Screen - Advanced Search<br>Database - CINAHL Complete | 2,233 |
| S11 | TI mental disorders OR AB mental disorders | Search modes - Boolean/Phrase | Interface - EBSCOhost Research Databases<br>Search Screen - Advanced Search<br>Database - CINAHL Complete | 23,954 |
| S10 | TI mental health risk OR AB mental health risk | Search modes - Boolean/Phrase | Interface - EBSCOhost Research Databases<br>Search Screen - Advanced Search<br>Database - CINAHL Complete | 5,622 |
| S9 | TI prevalence of mental health problems OR AB prevalence of mental health problems | Search modes - Boolean/Phrase | Interface - EBSCOhost Research Databases<br>Search Screen - Advanced Search<br>Database - CINAHL Complete | 612 |
| S8 | TI mental health issues OR AB mental health issues | Search modes - Boolean/Phrase | Interface - EBSCOhost Research Databases<br>Search Screen - Advanced Search<br>Database - CINAHL Complete | 6,002 |
| S7 | TI mental health problems OR AB mental | Search modes - Boolean/Phrase | Interface - EBSCOhost Research Databases | 14,297 |

| | | | | |
|---|---|---|---|---|
| | health problems | | Search Screen - Advanced Search<br>Database - CINAHL Complete | |
| S6 | TI mental health symptoms OR AB mental health symptoms | Search modes - Boolean/Phrase | Interface - EBSCOhost Research Databases<br>Search Screen - Advanced Search<br>Database - CINAHL Complete | 3,949 |
| S5 | TI mental health status OR AB mental health status | Search modes - Boolean/Phrase | Interface - EBSCOhost Research Databases<br>Search Screen - Advanced Search<br>Database - CINAHL Complete | 3,887 |
| S4 | TI mental wellbeing OR AB mental wellbeing | Search modes - Boolean/Phrase | Interface - EBSCOhost Research Databases<br>Search Screen - Advanced Search<br>Database - CINAHL Complete | 2,233 |
| S3 | TI mental health disorder* OR AB mental health disorder* | Search modes - Boolean/Phrase | Interface - EBSCOhost Research Databases<br>Search Screen - Advanced Search<br>Database - CINAHL Complete | 7,151 |
| S2 | TI mental distress OR AB mental distress | Search modes - Boolean/Phrase | Interface - EBSCOhost Research Databases<br>Search Screen - Advanced Search<br>Database - CINAHL Complete | 2,835 |
| S1 | TI mental health OR AB mental health | Search modes - Boolean/Phrase | Interface - EBSCOhost Research Databases<br>Search Screen - Advanced Search<br>Database - CINAHL Complete | 135,369 |
