# Supplementary material for: Mental health and mental health help-seeking behaviors among first-generation voluntary African migrants: A systematic review
Source: PLoS One. 2024 Mar 18;19(3):e0298634. doi: 10.1371/journal.pone.0298634 (PMC10947684; doi:10.1371/journal.pone.0298634)
Supplement: S2 Appendix — A. CINAHL Search Strategy 23.05.2023. B. Embase Search Strategy 23.05.2023. C. Medline Complete Search Strategy 23.05.2023. D. PsychInfo Search Strategy 23.05.2023. (ZIP) [file pone.0298634.s004.zip › S2B_Appendix.pdf]

TRY THE NEW OVID INTERFACE

Ovid®

Wolters Kluwer

▼ Search History (84)

View Saved

# ▼	Searches	Results	Type	Actions	Annotations
84	limit 83 to yr="2022 - 2023"	35	Advanced	Display Results | More	
83	40 and 61 and 78 and 82	273	Advanced	Display Results | More	
82	79 or 80 or 81	440941	Advanced	Display Results | More	
81	young adults.mp.	45287	Advanced	Display Results | More	
80	youth.mp.	118791	Advanced	Display Results | More	
79	adults.mp.	335319	Advanced	Display Results | More	
78	62 or 63 or 64 or 65 or 66 or 67 or 68 or 69 or 70 or 71 or 72 or 73 or 74 or 75 or 76 or 77	59503	Advanced	Display Results | More	
77	voluntary African migrants.mp.	1	Advanced	Display Results | More	
76	voluntary migrants.mp.	25	Advanced	Display Results | More	
75	first generation African immigrants.mp.	1	Advanced	Display Results | More	
74	first generation immigrants.mp.	357	Advanced	Display Results | More	
73	first generation migrants.mp.	88	Advanced	Display Results | More	
72	exp Minority Groups/ or minority population.mp.	21112	Advanced	Display Results | More	
71	people of colour.mp.	92	Advanced	Display Results | More	
70	foreigners.mp.	1005	Advanced	Display Results | More	
69	exp Immigration/	26640	Advanced	Display Results | More	
68	exp Immigration/	26640	Advanced	Display Results | More	
67	exp Undocumented Immigration/ or exp Immigration/	26640	Advanced	Display Results | More	
66	exp Migrant Workers/ or migrants.mp.	9077	Advanced	Display Results | More	
65	sub-Saharan African.mp.	915	Advanced	Display Results | More	
64	Africans in diaspora.mp.	0	Advanced	Save | More	
63	Africans.mp. or exp African Cultural Groups/	5525	Advanced	Display Results | More	
62	African migrants.mp.	148	Advanced	Display Results | More	
61	41 or 42 or 43 or 44 or 45 or 46 or 47 or 48 or 49 or 50 or 51 or 52 or 53 or 54 or 55 or 56 or 57 or 58 or 59 or 60	116059	Advanced	Display Results | More	
60	exp Health Literacy/	4400	Advanced	Display Results | More	
59	mental health information.mp.	382	Advanced	Display Results | More	
58	exp Mental Health Education/	6	Advanced	Display Results | More	

Contract

☐	57	exp Mental Health Literacy/	670	Advanced	Display Results | More
☐	56	coping skills.mp.	6578	Advanced	Display Results | More
☐	55	coping style.mp. or exp Coping Style/	5992	Advanced	Display Results | More
☐	54	coping mechanisms.mp.	3462	Advanced	Display Results | More
☐	53	coping strategies.mp.	23332	Advanced	Display Results | More
☐	52	support-seeking.mp.	1234	Advanced	Display Results | More
☐	51	mental health assistance.mp.	125	Advanced	Display Results | More
☐	50	mental help-seeking attitude.mp.	1	Advanced	Display Results | More
☐	49	mental health support.mp.	982	Advanced	Display Results | More
☐	48	helping behavior.mp. or exp "Assistance (Social Behavior)"/	56072	Advanced	Display Results | More
☐	47	help seeking support.mp.	6	Advanced	Display Results | More
☐	46	exp Social Support/ or exp Help Seeking Behavior/	59033	Advanced	Display Results | More
☐	45	help seeking behavior.mp. or exp Help Seeking Behavior/	16980	Advanced	Display Results | More
☐	44	help seeking behaviour.mp. [mp=title, abstract, heading word, table of contents, key concepts, original title, tests & measures, mesh word]	548	Advanced	Display Results | More
☐	43	help-seeking behavior.mp. [mp=title, abstract, heading word, table of contents, key concepts, original title, tests & measures, mesh word]	7701	Advanced	Display Results | More
☐	42	help-seeking behaviour.mp. [mp=title, abstract, heading word, table of contents, key concepts, original title, tests & measures, mesh word]	548	Advanced	Display Results | More
☐	41	help-seeking.mp. [mp=title, abstract, heading word, table of contents, key concepts, original title, tests & measures, mesh word]	12994	Advanced	Display Results | More
☐	40	1 or 2 or 3 or 4 or 5 or 6 or 7 or 8 or 9 or 10 or 11 or 12 or 13 or 14 or 15 or 16 or 17 or 18 or 19 or 20 or 21 or 22 or 23 or 24 or 25 or 26 or 27 or 28 or 29 or 30 or 31 or 32 or 33 or 34 or 35 or 36 or 37 or 38 or 39	1007120	Advanced	Display Results | More

☐	39	psychotic disorders.mp. [mp=title, abstract, heading word, table of contents, key concepts, original title, tests & measures, mesh word]	30409	Advanced	Display Results | More	
☐	38	psychiatric problems.mp. [mp=title, abstract, heading word, table of contents, key concepts, original title, tests & measures, mesh word]	3528	Advanced	Display Results | More	
☐	37	psychological distress.mp. [mp=title, abstract, heading word, table of contents, key concepts, original title, tests & measures, mesh word]	25208	Advanced	Display Results | More	
☐	36	psychological impact.mp. [mp=title, abstract, heading word, table of contents, key concepts, original title, tests & measures, mesh word]	3696	Advanced	Display Results | More	
☐	35	psychological problems.mp. [mp=title, abstract, heading word, table of contents, key concepts, original title, tests & measures, mesh word]	7677	Advanced	Display Results | More	
☐	34	physiological stress.mp. [mp=title, abstract, heading word, table of contents, key concepts, original title, tests & measures, mesh word]	4771	Advanced	Display Results | More	
☐	33	psychological stress.mp. [mp=title, abstract, heading word, table of contents, key concepts, original title, tests & measures, mesh word]	12976	Advanced	Display Results | More	
☐	32	mental fatigue.mp. [mp=title, abstract, heading word, table of contents, key concepts, original title, tests & measures, mesh word]	1333	Advanced	Display Results | More	
☐	31	tiredness.mp. [mp=title, abstract, heading word, table of contents, key concepts, original title, tests &	1605	Advanced	Display Results | More	

		measures, mesh word]			
30	chronic fatigue syndrome.mp. [mp=title, abstract, heading word, table of contents, key concepts, original title, tests & measures, mesh word]	2725	Advanced	Display Results | More	
29	chronic fatigue.mp. [mp=title, abstract, heading word, table of contents, key concepts, original title, tests & measures, mesh word]	3172	Advanced	Display Results | More	
28	fatigue.mp. [mp=title, abstract, heading word, table of contents, key concepts, original title, tests & measures, mesh word]	34337	Advanced	Display Results | More	
27	daytime sleepiness.mp. [mp=title, abstract, heading word, table of contents, key concepts, original title, tests & measures, mesh word]	3603	Advanced	Display Results | More	
26	sleeplessness.mp. [mp=title, abstract, heading word, table of contents, key concepts, original title, tests & measures, mesh word]	469	Advanced	Display Results | More	
25	insomnia.mp. [mp=title, abstract, heading word, table of contents, key concepts, original title, tests & measures, mesh word]	16518	Advanced	Display Results | More	
24	sleep problems.mp. [mp=title, abstract, heading word, table of contents, key concepts, original title, tests & measures, mesh word]	5438	Advanced	Display Results | More	
23	sleep disturbance.mp. [mp=title, abstract, heading word, table of contents, key concepts, original title, tests & measures, mesh word]	5760	Advanced	Display Results | More	
22	sleep disorders.mp. [mp=title, abstract, heading word, table of contents, key concepts, original title, tests & measures, mesh word]	8121	Advanced	Display Results | More	

21	post-traumatic stress disorder.mp. [mp=title, abstract, heading word, table of contents, key concepts, original title, tests & measures, mesh word]	14257	Advanced	Display Results | More	
20	stress disorder.mp. [mp=title, abstract, heading word, table of contents, key concepts, original title, tests & measures, mesh word]	52145	Advanced	Display Results | More	
19	stress.mp. [mp=title, abstract, heading word, table of contents, key concepts, original title, tests & measures, mesh word]	314566	Advanced	Display Results | More	
18	generalized anxiety disorder.mp. [mp=title, abstract, heading word, table of contents, key concepts, original title, tests & measures, mesh word]	12983	Advanced	Display Results | More	
17	anxiety anxiety disorder.mp. [mp=title, abstract, heading word, table of contents, key concepts, original title, tests & measures, mesh word]	16	Advanced	Display Results | More	
16	bipolar disorder.mp. [mp=title, abstract, heading word, table of contents, key concepts, original title, tests & measures, mesh word]	45112	Advanced	Display Results | More	
15	seasonal affective disorder.mp. [mp=title, abstract, heading word, table of contents, key concepts, original title, tests & measures, mesh word]	1647	Advanced	Display Results | More	
14	depressive disorder.mp. [mp=title, abstract, heading word, table of contents, key concepts, original title, tests & measures, mesh word]	79040	Advanced	Display Results | More	
13	depression.mp. [mp=title, abstract, heading word, table of contents, key concepts, original title, tests & measures, mesh word]	382663	Advanced	Display Results | More	

12	mental wellbeing.mp. [mp=title, abstract, heading word, table of contents, key concepts, original title, tests & measures, mesh word]	1038	Advanced	Display Results | More	
11	mental disorders.mp. [mp=title, abstract, heading word, table of contents, key concepts, original title, tests & measures, mesh word]	191904	Advanced	Display Results | More	
10	mental health risk.mp. [mp=title, abstract, heading word, table of contents, key concepts, original title, tests & measures, mesh word]	434	Advanced	Display Results | More	
9	prevalence of mental health problems.mp. [mp=title, abstract, heading word, table of contents, key concepts, original title, tests & measures, mesh word]	356	Advanced	Display Results | More	
8	mental health issues.mp. [mp=title, abstract, heading word, table of contents, key concepts, original title, tests & measures, mesh word]	6082	Advanced	Display Results | More	
7	mental health problems.mp. [mp=title, abstract, heading word, table of contents, key concepts, original title, tests & measures, mesh word]	18693	Advanced	Display Results | More	
6	mental health symptoms.mp. [mp=title, abstract, heading word, table of contents, key concepts, original title, tests & measures, mesh word]	3145	Advanced	Display Results | More	
5	mental health status.mp. [mp=title, abstract, heading word, table of contents, key concepts, original title, tests & measures, mesh word]	3367	Advanced	Display Results | More	
4	mental wellbeing.mp. [mp=title, abstract, heading word, table of contents, key concepts, original title, tests & measures, mesh word]	1038	Advanced	Display Results | More	

☐	3	mental health disorder.mp. [mp=title, abstract, heading word, table of contents, key concepts, original title, tests & measures, mesh word]	961	Advanced	Display Results | More	
☐	2	mental distress.mp. [mp=title, abstract, heading word, table of contents, key concepts, original title, tests & measures, mesh word]	2317	Advanced	Display Results | More	
☐	1	Mental health.mp. [mp=title, abstract, heading word, table of contents, key concepts, original title, tests & measures, mesh word]	279269	Advanced	Display Results | More	

Save Remove Combine with: AND OR

Save All Edit Create RSS Create Auto-Alert View Saved

Email All Search History Copy Search History Link Copy Search History Details

Basic Search | Find Citation | Search Tools | Search Fields | **Advanced Search** | Multi-Field Search

1 Resource selected | Hide | Change

APA PsycInfo 1806 to May Week 3 2023

◉ **Keyword** ○ Author ○ Title ○ Journal

Enter keyword or phrase
(* or $ for truncation)

Expand Term Finder ▸

Search

☐ Include Multimedia
☑ Map Term to Subject Heading

▾ **Limits** *(close)*

☐ Full Text
☐ APA PsycArticles Journals
☐ All Journals
☐ Latest Update
☐ Human
☐ English Language
☐ Abstracts
☐ Test DOI
☐ Open Access
☐ Impact Statement

Publication Year
- - -

Additional Limits Edit Limits

To search Open Access content on Ovid, go to Basic Search.

Options ◂

Search Information

You searched:

limit 83 to yr="2022 - 2023"

Search terms used:

adults
african
cultural
groups
migrants
africans
in
diaspora
anxiety
disorder
assistance
(social
behavior)
bipolar
chronic
fatigue
syndrome
coping
mechanisms
skills
strategies
style
daytime
sleepiness
depression
depressive
first
generation
immigrants
foreigners
generalized
health
literacy
help
seeking
behavior
behaviour
support
help-seeking
helping
immigration
insomnia
mental
disorders
distress
education
information
issues
problems
risk
status
symptoms
attitude
wellbeing
migrant
workers
minority
population
people
of
colour
physiological
stress
post-traumatic
prevalence
psychiatric
psychological
impact
psychotic
seasonal
affective
sleep
disturbance
sleeplessness
social

sub-saharan
support-seeking
tiredness
undocumented
voluntary
young
youth

Search Returned:
35 text results

Sort By:
-

Customize Display

Filter By

Add to Search History

Selected Only (0)

▼ Years

All Years
Current year
Past 3 years
Past 5 years
▶ Specific Year Range

▶ Subject
▶ Author
▶ Journal
▶ Book
▶ Publication Type

My Projects

+ New Project

No projects available.

JBI EBP Tools

SUMARI

PACES

Print Email Export + My Projects Keep Selected

All Range

Clear

50 Per Page

1.

Patient empowerment among transgender and gender diverse youth. [References].

Pflugeisen, Chaya Mangel; Boomgaarden, Anna; Denaro, Aytch A; Konicek, Danielle; Robinson, Emily.

LGBT Health. 2023, pp. No Pagination Specified.

[Journal; Peer Reviewed Journal]

Year of Publication

2023

Publication Month/Season

May

Abstract
Cite + My Projects
+ Annotate
Annotation(s)

Abstract Reference
Complete Reference

Find Similar
Find Citing Articles

Find It @ UniMelb

2.
The impact of parent support on patient empowerment in trans and gender diverse youth. [References].

Pflugeisen, Chaya Mangel; Denaro, Aytch A; Boomgaarden, Anna.

LGBTQ+ Family: An Interdisciplinary Journal. 2023, pp. No Pagination Specified.

[Journal; Peer Reviewed Journal]

Year of Publication

2023

Publication Month/Season

Apr

Abstract
Cite + My Projects
+ Annotate
Annotation(s)

Abstract Reference
Complete Reference

Find Similar
Find Citing Articles

Find It @ UniMelb

3.
Factors associated with racial and ethnic minority youths' mental health help-seeking at school.

Allouche, Sam.

Dissertation Abstracts International: Section B: The Sciences and Engineering. Vol.84(4-B), 2023, pp. No Pagination Specified.

[Dissertation Abstract]

Year of Publication

2023

Abstract
Cite + My Projects
+ Annotate
Annotation(s)

Abstract Reference
Complete Reference

Find Similar
Find Citing Articles

Find It @ UniMelb

4.
Construct validity of the Enfranchisement Scale of the Community Participation Indicators. [References].

Kersey, Jessica; Terhorst, Lauren; Heinemann, Allen W; Hammel, Joy; Baum, Carolyn; McCue, Michael; Skidmore, Elizabeth R.

Clinical Rehabilitation. Vol.36(2), 2022, pp. 263-271.

[Journal; Peer Reviewed Journal]

Year of Publication

2022

Publication Month/Season

Feb

Abstract

Cite + My Projects
+ Annotate
Annotation(s)

Abstract Reference
Complete Reference

Find Similar
Find Citing Articles

Find It @ UniMelb

5.
Adverse childhood experiences and alcohol use among u.S.-born and immigrant latinx youth: The roles of social support and stress hormones. [References].

Zhen-Duan, Jenny; Nunez, Miguel; Solomon, Matia B; Geracioti, Thomas; Jacquez, Farrah.

Journal of Child and Family Studies. 2023, pp. No Pagination Specified.

[Journal; Peer Reviewed Journal]

Year of Publication

2023

Publication Month/Season

Feb

Abstract
Cite + My Projects
+ Annotate
Annotation(s)

Abstract Reference
Complete Reference

Find Similar
Find Citing Articles

Find It @ UniMelb

6.
Exploring mental health and help-seeking attitudes among sexual minoritized adults in Utah. [References].

McGraw, James S; Oakey-Frost, D. Nicolas; Lefevor, G. Tyler; Docherty, Meagan; Tucker, Raymond P.

Psychology of Sexual Orientation and Gender Diversity. 2023, pp. No Pagination Specified.

[Journal; Peer Reviewed Journal]

Year of Publication

2023

Publication Month/Season

Mar

Abstract
Cite + My Projects
+ Annotate
Annotation(s)

Abstract Reference
Complete Reference

Find Similar
Find Citing Articles

Find It @ UniMelb

7.
The longitudinal impact of discrimination on attention problems in Latinx immigrant youth: Examining the roles of somatic symptoms and social support.

Kuperman, Kelsey L.

Dissertation Abstracts International: Section B: The Sciences and Engineering. Vol.84(4-B), 2023, pp. No Pagination Specified.

[Dissertation Abstract]

Year of Publication

2023

Abstract
Cite + My Projects
+ Annotate
Annotation(s)

Abstract Reference
Complete Reference

Find Similar
Find Citing Articles

Find It @ UniMelb

8.
Handbook of social inclusion: Research and practices in health and social sciences.

Liamputtong, Pranee [Ed].

(2022). Handbook of social inclusion: Research and practices in health and social sciences. lxxiv, 2317 pp. Cham, Switzerland: Springer Nature Switzerland AG; Switzerland.

[Book; Edited Book]

Year of Publication

2022

Abstract
Cite + My Projects
+ Annotate
Annotation(s)

Abstract Reference
Complete Reference

Find Similar
Find Citing Articles

Find It @ UniMelb

9.
Optimizing the design and implementation of peer support interventions for adolescents living with HIV in Sub-Saharan Africa.

Ahmed, Charisse Victoria.

Dissertation Abstracts International: Section B: The Sciences and Engineering. Vol.84(3-B), 2023, pp. No Pagination Specified.

[Dissertation Abstract]

Year of Publication

2023

Abstract
Cite + My Projects
+ Annotate
Annotation(s)

Abstract Reference
Complete Reference

Find Similar
Find Citing Articles

Find It @ UniMelb

☐10.

Determinants of mental health in the context of multiple minority status: An examination of Muslim American young adults.

Saifan, Dana.

Dissertation Abstracts International: Section B: The Sciences and Engineering. Vol.84(2-B), 2023, pp. No Pagination Specified.

[Dissertation Abstract]

Year of Publication

2023

Abstract
Cite + My Projects
+ Annotate
Annotation(s)

Abstract Reference
Complete Reference

Find Similar
Find Citing Articles

Find It @ UniMelb

☐11.

Youth mental health first aid for educators of immigrant-origin youth: A mixed-method evaluation of the virtual delivery approach.

Chooi, Olivia Khoo Kit.

Dissertation Abstracts International: Section B: The Sciences and Engineering. Vol.84(1-B), 2023, pp. No Pagination Specified.

[Dissertation Abstract]

Year of Publication

2023

Abstract
Cite + My Projects
+ Annotate
Annotation(s)

Abstract Reference
Complete Reference

Find Similar
Find Citing Articles

Find It @ UniMelb

☐12.

The disproportionate impact of COVID-19 on minority groups: A social justice concern. [References].

Lee, HeeSoon; Miller, Vivian J.

Putnam, Michelle [Ed]; Shen, Huei-Wern [Ed]. (2022). Gerontological social work and COVID-19: Calls for change in education, practice, and policy from international voices. (pp. 87-91). xxii, 249 pp. New York, NY, US: Routledge/Taylor & Francis Group; US.

[Book; Edited Book]

Year of Publication

2022

Abstract
Cite + My Projects
+ Annotate
Annotation(s)

Abstract Reference
Complete Reference

Find Similar
Find Citing Articles

Find It @ UniMelb

13.

Teaching DBT skills to DACA recipients and their families: Findings from an ECHO program. [References].

Morales, Frances R; Rojas Perez, Oscar F; Silva, Michelle A; Paris, Manuel Jr.; Garcini, Luz M; Domenech Rodriguez, Melanie M; Mercado, Alfonso.

Practice Innovations. Vol.7(4), 2022, pp. 327-341.

[Journal; Peer Reviewed Journal]

Year of Publication

2022

Publication Month/Season

Dec

Abstract
Article as PDF (404KB)
Cite
+ My Projects
+ Annotate
Annotation(s)

Ovid Full Text
Abstract Reference
Complete Reference

Find Similar
Find Citing Articles

14.

Self-esteem in sexual minority young adults: A qualitative interview study exploring protective factors and helpful coping responses. [References].

Bridge, Livia; Smith, Patrick; Rimes, Katharine A.

International Review of Psychiatry. Vol.34(3-4), 2022, pp. 257-265.

[Journal; Peer Reviewed Journal]

Year of Publication

2022

Publication Month/Season

May-Jun

Abstract
Cite
+ My Projects
+ Annotate
Annotation(s)

Abstract Reference
Complete Reference

Find Similar
Find Citing Articles

Find It @ UniMelb

15.

Asian American adolescents' mental health literacy and beliefs about helpful strategies to address mental health challenges at school. [References].

Liu, Jia Li; Wang, Cixin; Do, Kieu Anh; Bali, Diksha.

Psychology in the Schools. Vol.59(10), 2022, pp. 2062-2084.

[Journal; Peer Reviewed Journal]

Year of Publication

2022

Publication Month/Season

Oct

Abstract
Cite + My Projects
+ Annotate
Annotation(s)

Abstract Reference
Complete Reference

Find Similar
Find Citing Articles

Find It @ UniMelb

16.
Stress and resilience among resettling refugee youth: An illustrative review and new applications for the family stress model. [References].

Masarik, April S; Fritz, Hailey; Lazarevic, Vanja.

Journal of Family Theory & Review. Vol.14(2), 2022, pp. 207-232.

[Journal; Peer Reviewed Journal]

Year of Publication

2022

Publication Month/Season

Jun

Abstract
Cite + My Projects
+ Annotate
Annotation(s)

Abstract Reference
Complete Reference

Find Similar
Find Citing Articles

Find It @ UniMelb

17.
Taking a paradoxical and physiological approach to Cardona, Madigan, and Sauer-Zavela's conceptualization of chronic, traumatic invalidation as a primary factor in the relationship between minority stress and disproportionate health burden among sexual and gender minority adults. [References].

Hillman, Jennifer.

Clinical Psychology: Science and Practice. Vol.29(2), 2022, pp. 196-199.

[Journal; Peer Reviewed Journal]

Year of Publication

2022

Publication Month/Season

Jun

Abstract
Article as PDF (91KB)
Cite
+ My Projects
+ Annotate
Annotation(s)

Ovid Full Text
Abstract Reference
Complete Reference

Find Similar

Find Citing Articles

18.

Mental health among LGBT youth. [References].

Choukas-Bradley, Sophia; Thoma, Brian C.

VanderLaan, Doug P [Ed]; Wong, Wang Ivy [Ed]. (2022). Gender and sexuality development: Contemporary theory and research. (pp. 539-565). xxiii, 636 pp. Cham, Switzerland: Springer Nature Switzerland AG; Switzerland.

[Book; Edited Book]

Year of Publication

2022

Abstract
Cite + My Projects
+ Annotate
Annotation(s)

Abstract Reference
Complete Reference

Find Similar
Find Citing Articles

Find It @ UniMelb

19.

Unaccompanied migrant children in the United States: Implementation and effectiveness of trauma-focused cognitive behavioral therapy. [References].

Patel, Zabin S; Casline, Elizabeth P; Vera, Cedrin; Ramirez, Vanessa; Jensen-Doss, Amanda.

Psychological Trauma: Theory, Research, Practice, and Policy. 2022, pp. No Pagination Specified.

[Journal; Peer Reviewed Journal]

Year of Publication

2022

Publication Month/Season

Sep

Abstract
Cite + My Projects
+ Annotate
Annotation(s)

Abstract Reference
Complete Reference

Find Similar
Find Citing Articles

Find It @ UniMelb

20.

Policy stress and social support: Mental health impacts for Latinx Adults in the Southeast United States. [References].

Held, Mary Lehman; First, Jennifer M; Huslage, Melody; Holzer, Marie.

Social Science & Medicine. Vol.307 2022, pp. 1-9. ArtID 115172.

[Journal; Peer Reviewed Journal]

Year of Publication

2022

Publication Month/Season

Aug

Abstract

Cite + My Projects
+ Annotate
Annotation(s)

Abstract Reference
Complete Reference

Find Similar
Find Citing Articles

Find It @ UniMelb

21.
Disordered eating across COVID-19 in LGBTQ+ young adults. [References].

Hart, Erica A; Rubin, Alex; Kline, Kiki M; Fox, Kathryn R.

Eating Behaviors. Vol.44 2022, pp. 1-6. ArtID 101581.

[Journal; Peer Reviewed Journal]

Year of Publication

2022

Publication Month/Season

Jan

Abstract
Cite + My Projects
+ Annotate
Annotation(s)

Abstract Reference
Complete Reference

Find Similar
Find Citing Articles

Find It @ UniMelb

22.
Understanding protective factors for suicidality and depression among U.S. Sexual and gender minority adolescents: Implications for school psychologists. [References].

Rivas-Koehl, Matthew; Valido, Alberto; Espelage, Dorothy L; Robinson, Luz E; Hong, Jun Sung; Kuehl, Tomei; Mintz, Sasha; Wyman, Peter A.

School Psychology Review. Vol.51(3), 2022, pp. 290-303.

[Journal; Peer Reviewed Journal]

Year of Publication

2022

Abstract
Cite + My Projects
+ Annotate
Annotation(s)

Abstract Reference
Complete Reference

Find Similar
Find Citing Articles

Find It @ UniMelb

23.
The quality of life and well-being among older immigrants of Eastern European origin.

Stadnik, Natalia.

Dissertation Abstracts International: Section B: The Sciences and Engineering. Vol.83(9-B), 2022, pp. No Pagination Specified.

[Dissertation Abstract]

Year of Publication

2022

Abstract
Cite + My Projects
+ Annotate
Annotation(s)

Abstract Reference
Complete Reference

Find Similar
Find Citing Articles

Find It @ UniMelb

24.
"It doesn't matter how good the school is if you don't learn to socialize": Latinx immigrant students' testimonios of coping with social isolation in high school. [References].

Lilly, Jenn M.

Children and Youth Services Review. Vol.137 2022, pp. 1-9. ArtID 106476.

[Journal; Peer Reviewed Journal]

Year of Publication

2022

Publication Month/Season

Jun

Abstract
Cite + My Projects
+ Annotate
Annotation(s)

Abstract Reference
Complete Reference

Find Similar
Find Citing Articles

Find It @ UniMelb

25.
Identity-specific positive psychology intervention for sexual minorities: A randomized control trial.

Job, Sarah A.

Dissertation Abstracts International Section A: Humanities and Social Sciences. Vol.83(5-A), 2022, pp. No Pagination Specified.

[Dissertation Abstract]

Year of Publication

2022

Abstract
Cite + My Projects
+ Annotate
Annotation(s)

Abstract Reference
Complete Reference

Find Similar
Find Citing Articles

Find It @ UniMelb

☐26.

Examining homeless trajectories and health outcomes among young adults in Los Angeles County.

Richards, Jessica Kathryn.

Dissertation Abstracts International: Section B: The Sciences and Engineering. Vol.83(4-B), 2022, pp. No Pagination Specified.

[Dissertation Abstract]

Year of Publication

2022

Abstract
Cite + My Projects
+ Annotate
Annotation(s)

Abstract Reference
Complete Reference

Find Similar
Find Citing Articles

Find It @ UniMelb

☐27.

An exploration of social media as a useful resource for sexual minorities during emerging adulthood.

Kaviani, Kimia.

Dissertation Abstracts International: Section B: The Sciences and Engineering. Vol.83(4-B), 2022, pp. No Pagination Specified.

[Dissertation Abstract]

Year of Publication

2022

Abstract
Cite + My Projects
+ Annotate
Annotation(s)

Abstract Reference
Complete Reference

Find Similar
Find Citing Articles

Find It @ UniMelb

28.

Development of a minority stress preventive intervention for sexual and gender minority youth and young adults.

Holt, Natalie R.

Dissertation Abstracts International: Section B: The Sciences and Engineering. Vol.83(2-B), 2022, pp. No Pagination Specified.

[Dissertation Abstract]

Year of Publication

2022

Abstract
Cite + My Projects
+ Annotate
Annotation(s)

Abstract Reference
Complete Reference

Find Similar
Find Citing Articles

Find It @ UniMelb

29.

The role of trajectories of stress and social support in underrepresented students' educational outcomes. [References].

Wittrup, Audrey R; Hurd, Noelle M.

Applied Developmental Science. Vol.26(3), 2022, pp. 532-552.

[Journal; Peer Reviewed Journal]

Year of Publication

2022

Publication Month/Season

Jul-Sep

Abstract
Cite + My Projects
+ Annotate
Annotation(s)

Abstract Reference
Complete Reference

Find Similar
Find Citing Articles

Find It @ UniMelb

30.

The association between daily concealment and affect among sexual and gender minority adolescents: The moderating role of family and peer support. [References].

Kiekens, Wouter J; Mereish, Ethan H.

Journal of Adolescent Health. Vol.70(4), 2022, pp. 650-657.

[Journal; Peer Reviewed Journal]

Year of Publication

2022

Publication Month/Season

Apr

Abstract
Cite + My Projects
+ Annotate
Annotation(s)

Abstract Reference

Complete Reference

Find Similar
Find Citing Articles

Find It @ UniMelb

31.
Perceived discrimination, coping styles, and internalizing symptoms among a community sample of Hispanic and Somali adolescents. [References].

Forster, Myriam; Grigsby, Timothy; Rogers, Christopher; Unger, Jennifer; Alvarado, Stephanie; Rainisch, Bethany; Areba, Eunice.

Journal of Adolescent Health. Vol.70(3), 2022, pp. 488-495.

[Journal; Peer Reviewed Journal]

Year of Publication

2022

Publication Month/Season

Mar

Abstract
Cite + My Projects
+ Annotate
Annotation(s)

Abstract Reference
Complete Reference

Find Similar
Find Citing Articles

Find It @ UniMelb

32.
The relations between the positive and negative components of self-compassion and depressive symptoms among sexual minority women and men. [References].

Shakeshaft, Rhianydd; McLaren, Suzanne.

Mindfulness. Vol.13(1), 2022, pp. 57-65.

[Journal; Peer Reviewed Journal]

Year of Publication

2022

Publication Month/Season

Jan

Abstract
Cite + My Projects
+ Annotate
Annotation(s)

Abstract Reference
Complete Reference

Find Similar
Find Citing Articles

Find It @ UniMelb

33.
Understanding unaccompanied immigrant youth's experiences in US schools: An interdisciplinary perspective. [References].

Frankel, Katherine K; Brabeck, Kalina M; Rendon Garcia, Sarah A.

Journal of Education for Students Placed at Risk. Vol.27(1), 2022, pp. 27-58.

[Journal; Peer Reviewed Journal]

Year of Publication

2022

Publication Month/Season

Jan-Mar

Abstract
Cite + My Projects
+ Annotate
Annotation(s)

Abstract Reference
Complete Reference

Find Similar
Find Citing Articles

Find It @ UniMelb

34.
Understanding the migration experience of unaccompanied youth: A review of the literature. [References].

Garcia, Maria Fernanda; Birman, Dina.

American Journal of Orthopsychiatry. Vol.92(1), 2022, pp. 79-102.

[Journal; Peer Reviewed Journal]

Year of Publication

2022

Abstract
Article as PDF (542KB)
Cite
+ My Projects
+ Annotate
Annotation(s)

Ovid Full Text
Abstract Reference
Complete Reference

Find Similar
Find Citing Articles

35.
Sexual orientation, social support, and mental health resilience in a U.S. national sample of adults. [References].

Krueger, Evan A; Upchurch, Dawn M.

Behavioral Medicine. Vol.48(3), 2022, pp. 207-215.

[Journal; Peer Reviewed Journal]

Year of Publication

2022

Publication Month/Season

Jul-Sep

Abstract
Cite + My Projects
+ Annotate
Annotation(s)

Abstract Reference
Complete Reference

Find Similar
Find Citing Articles

Find It @ UniMelb

All Range

Clear

50 Per Page

Print Email Export + My Projects Keep Selected

English Français Italiano Deutsch 日本語 繁體中文 Español 简体中文 한국어

About Us Contact Us Privacy Policy Terms of Use

© 2023 Ovid Technologies, Inc. All rights reserved. OvidUI_04.26.00.015, SourceID e55a8f503493ed5a427d7dad41b71879279ee8a7
