# Supplementary material for: Mental health and mental health help-seeking behaviors among first-generation voluntary African migrants: A systematic review
Source: PLoS One. 2024 Mar 18;19(3):e0298634. doi: 10.1371/journal.pone.0298634 (PMC10947684; doi:10.1371/journal.pone.0298634)
Supplement: S2 Appendix — A. CINAHL Search Strategy 23.05.2023. B. Embase Search Strategy 23.05.2023. C. Medline Complete Search Strategy 23.05.2023. D. PsychInfo Search Strategy 23.05.2023. (ZIP) [file pone.0298634.s004.zip › S2D_Appendix.pdf]

Database(s): **APA PsycInfo** 1806 to May Week 3 2023

1.
Patient empowerment among transgender and gender diverse **youth**. [References].

Pflugeisen, Chaya Mangel; Boomgaarden, Anna; Denaro, Aytch A; Konicek, Danielle; Robinson, Emily.

LGBT Health. 2023, pp. No Pagination Specified.

[Journal; Peer Reviewed Journal]

Year of Publication

2023

Publication Month/Season

May

Cite

2.
The **impact of** parent **support** on patient empowerment **in** trans and gender diverse **youth**. [References].

Pflugeisen, Chaya Mangel; Denaro, Aytch A; Boomgaarden, Anna.

LGBTQ+ Family: An Interdisciplinary Journal. 2023, pp. No Pagination Specified.

[Journal; Peer Reviewed Journal]

Year of Publication

2023

Publication Month/Season

Apr

Cite

3.
Factors associated with racial and ethnic **minority** youths' **mental health help-seeking** at school.

Allouche, Sam.

Dissertation Abstracts International: Section B: The Sciences and Engineering. Vol.84(4-B), 2023, pp. No Pagination Specified.

[Dissertation Abstract]

Year of Publication

2023

Cite

4.
Construct validity **of** the Enfranchisement Scale **of** the Community Participation Indicators. [References].

Kersey, Jessica; Terhorst, Lauren; Heinemann, Allen W; Hammel, Joy; Baum, Carolyn; McCue, Michael; Skidmore, Elizabeth R.

Clinical Rehabilitation. Vol.36(2), 2022, pp. 263-271.

[Journal; Peer Reviewed Journal]

Year of Publication

2022

Publication Month/Season

Feb

Cite

5.
Adverse childhood experiences and alcohol use among u.S.-born and immigrant latinx **youth**: The roles **of** social **support** and **stress** hormones. [References].

Zhen-Duan, Jenny; Nunez, Miguel; Solomon, Matia B; Geracioti, Thomas; Jacquez, Farrah.

Journal of Child and Family Studies. 2023, pp. No Pagination Specified.

[Journal; Peer Reviewed Journal]

Year of Publication

2023

Publication Month/Season

Feb

Cite

6.
Exploring **mental health** and **help-seeking** attitudes among sexual minoritized **adults in** Utah. [References].

McGraw, James S; Oakey-Frost, D. Nicolas; Lefevor, G. Tyler; Docherty, Meagan; Tucker, Raymond P.

Psychology of Sexual Orientation and Gender Diversity. 2023, pp. No Pagination Specified.

[Journal; Peer Reviewed Journal]

Year of Publication

2023

Publication Month/Season

Mar

Cite

7.
The longitudinal **impact of** discrimination on attention **problems in** Latinx immigrant **youth**: Examining the roles **of** somatic **symptoms** and social **support**.

Kuperman, Kelsey L.

Dissertation Abstracts International: Section B: The Sciences and Engineering. Vol.84(4-B), 2023, pp. No Pagination Specified.

[Dissertation Abstract]

Year of Publication

2023

Cite

8.
Handbook **of** social inclusion: Research and practices **in health** and social sciences.

Liamputtong, Pranee [Ed].

(2022). Handbook of social inclusion: Research and practices in health and social sciences. lxxiv, 2317 pp. Cham, Switzerland: Springer Nature Switzerland AG; Switzerland.

[Book; Edited Book]

Year of Publication

2022

Cite

9.
Optimizing the design and implementation **of** peer **support** interventions for adolescents living with HIV **in Sub-Saharan** Africa.

Ahmed, Charisse Victoria.

Dissertation Abstracts International: Section B: The Sciences and Engineering. Vol.84(3-B), 2023, pp. No Pagination Specified.

[Dissertation Abstract]

Year of Publication

2023

Cite

10.
Determinants **of mental health in** the context **of** multiple **minority status**: An examination **of** Muslim American **young adults**.

Saifan, Dana.

Dissertation Abstracts International: Section B: The Sciences and Engineering. Vol.84(2-B), 2023, pp. No Pagination Specified.

[Dissertation Abstract]

Year of Publication

2023

Cite

11.
Youth mental health first aid for educators **of** immigrant-origin **youth**: A mixed-method evaluation **of** the virtual delivery approach.

Chooi, Olivia Khoo Kit.

Dissertation Abstracts International: Section B: The Sciences and Engineering. Vol.84(1-B), 2023, pp. No Pagination Specified.

[Dissertation Abstract]

Year of Publication

2023

Cite

12.
The disproportionate **impact of** COVID-19 on **minority** groups: A social justice concern. [References].

Lee, HeeSoon; Miller, Vivian J.

Putnam, Michelle [Ed]; Shen, Huei-Wern [Ed]. (2022). Gerontological social work and COVID-19: Calls for change in education, practice, and policy from international voices. (pp. 87-91). xxii, 249 pp. New York, NY, US: Routledge/Taylor & Francis Group; US.

[Book; Edited Book]

Year of Publication

2022

Cite

13.
Teaching DBT **skills** to DACA recipients and their families: Findings from an ECHO program. [References].

Morales, Frances R; Rojas Perez, Oscar F; Silva, Michelle A; Paris, Manuel Jr.; Garcini, Luz M; Domenech Rodriguez, Melanie M; Mercado, Alfonso.

Practice Innovations. Vol.7(4), 2022, pp. 327-341.

[Journal; Peer Reviewed Journal]

Year of Publication

2022

Publication Month/Season

Dec

Cite

14.
Self-esteem **in** sexual **minority young adults**: A qualitative interview study exploring protective factors and helpful **coping** responses. [References].

Bridge, Livia; Smith, Patrick; Rimes, Katharine A.

International Review of Psychiatry. Vol.34(3-4), 2022, pp. 257-265.

[Journal; Peer Reviewed Journal]

Year of Publication

2022

Publication Month/Season

May-Jun

Cite

15.
Asian American adolescents' **mental health** literacy and beliefs about helpful **strategies** to address **mental health** challenges at school. [References].

Liu, Jia Li; Wang, Cixin; Do, Kieu Anh; Bali, Diksha.

Psychology in the Schools. Vol.59(10), 2022, pp. 2062-2084.

[Journal; Peer Reviewed Journal]

Year of Publication

2022

Publication Month/Season

Oct

Cite

16.
Stress and resilience among resettling refugee **youth**: An illustrative review and new applications for the family **stress** model. [References].

Masarik, April S; Fritz, Hailey; Lazarevic, Vanja.

Journal of Family Theory & Review. Vol.14(2), 2022, pp. 207-232.

[Journal; Peer Reviewed Journal]

Year of Publication

2022

Publication Month/Season

Jun

Cite

17.
Taking a paradoxical and **physiological** approach to Cardona, Madigan, and Sauer-Zavela's conceptualization **of chronic**, traumatic invalidation as a primary factor **in** the relationship between **minority stress** and disproportionate **health** burden among sexual and gender **minority adults**. [References].

Hillman, Jennifer.

Clinical Psychology: Science and Practice. Vol.29(2), 2022, pp. 196-199.

[Journal; Peer Reviewed Journal]

Year of Publication

2022

Publication Month/Season

Jun

Cite

18.
Mental health among LGBT **youth**. [References].

Choukas-Bradley, Sophia; Thoma, Brian C.

VanderLaan, Doug P [Ed]; Wong, Wang Ivy [Ed]. (2022). Gender and sexuality development: Contemporary theory and research. (pp. 539-565). xxiii, 636 pp. Cham, Switzerland: Springer Nature Switzerland AG; Switzerland.

[Book; Edited Book]

Year of Publication

2022

Cite

19.
Unaccompanied migrant children **in** the United States: Implementation and effectiveness **of** trauma-focused cognitive behavioral therapy. [References].

Patel, Zabin S; Casline, Elizabeth P; Vera, Cedrin; Ramirez, Vanessa; Jensen-Doss, Amanda.

Psychological Trauma: Theory, Research, Practice, and Policy. 2022, pp. No Pagination Specified.

[Journal; Peer Reviewed Journal]

Year of Publication

2022

Publication Month/Season

Sep

Cite

20.
Policy **stress** and social **support**: **Mental health** impacts for Latinx **Adults in** the Southeast United States. [References].

Held, Mary Lehman; First, Jennifer M; Huslage, Melody; Holzer, Marie.

Social Science & Medicine. Vol.307 2022, pp. 1-9. ArtID 115172.

[Journal; Peer Reviewed Journal]

Year of Publication

2022

Publication Month/Season

Aug

Cite

21.
Disordered eating across COVID-19 **in** LGBTQ+ **young adults**. [References].

Hart, Erica A; Rubin, Alex; Kline, Kiki M; Fox, Kathryn R.

Eating Behaviors. Vol.44 2022, pp. 1-6. ArtID 101581.

[Journal; Peer Reviewed Journal]

Year of Publication

2022

Publication Month/Season

Jan

Cite

22.
Understanding protective factors for suicidality and **depression** among U.S. Sexual and gender **minority** adolescents: Implications for school psychologists. [References].

Rivas-Koehl, Matthew; Valido, Alberto; Espelage, Dorothy L; Robinson, Luz E; Hong, Jun Sung; Kuehl, Tomei; Mintz, Sasha; Wyman, Peter A.

School Psychology Review. Vol.51(3), 2022, pp. 290-303.

[Journal; Peer Reviewed Journal]

Year of Publication

2022

Cite

23.
The quality **of** life and well-being among older **immigrants of** Eastern European origin.

Stadnik, Natalia.

Dissertation Abstracts International: Section B: The Sciences and Engineering. Vol.83(9-B), 2022, pp. No Pagination Specified.

[Dissertation Abstract]

Year of Publication

2022

Cite

24.
"It doesn't matter how good the school is if you don't learn to socialize": Latinx immigrant students' testimonios **of coping** with social isolation **in** high school. [References].

Lilly, Jenn M.

Children and Youth Services Review. Vol.137 2022, pp. 1-9. ArtID 106476.

[Journal; Peer Reviewed Journal]

Year of Publication

2022

Publication Month/Season

Jun

Cite

25.
Identity-specific positive psychology intervention for sexual minorities: A randomized control trial.

Job, Sarah A.

Dissertation Abstracts International Section A: Humanities and Social Sciences. Vol.83(5-A), 2022, pp. No Pagination Specified.

[Dissertation Abstract]

Year of Publication

2022

Cite

26.
Examining homeless trajectories and **health** outcomes among **young adults in** Los Angeles County.

Richards, Jessica Kathryn.

Dissertation Abstracts International: Section B: The Sciences and Engineering. Vol.83(4-B), 2022, pp. No Pagination Specified.

[Dissertation Abstract]

Year of Publication

2022

Cite

27.
An exploration **of** social media as a useful resource for sexual minorities during emerging adulthood.

Kaviani, Kimia.

Dissertation Abstracts International: Section B: The Sciences and Engineering. Vol.83(4-B), 2022, pp. No Pagination Specified.

[Dissertation Abstract]

Year of Publication

2022

Cite

28.
Development **of** a **minority stress** preventive intervention for sexual and gender **minority youth** and **young adults**.

Holt, Natalie R.

Dissertation Abstracts International: Section B: The Sciences and Engineering. Vol.83(2-B), 2022, pp. No Pagination Specified.

[Dissertation Abstract]

Year of Publication

2022

Cite

29.
The role **of** trajectories **of stress** and social **support in** underrepresented students' educational outcomes. [References].

Wittrup, Audrey R; Hurd, Noelle M.

Applied Developmental Science. Vol.26(3), 2022, pp. 532-552.

[Journal; Peer Reviewed Journal]

Year of Publication

2022

Publication Month/Season

Jul-Sep

Cite

30.
The association between daily concealment and affect among sexual and gender **minority** adolescents: The moderating role **of** family and peer **support**. [References].

Kiekens, Wouter J; Mereish, Ethan H.

Journal of Adolescent Health. Vol.70(4), 2022, pp. 650-657.

[Journal; Peer Reviewed Journal]

Year of Publication

2022

Publication Month/Season

Apr

Cite

31.
Perceived discrimination, **coping** styles, and internalizing **symptoms** among a community sample **of** Hispanic and Somali adolescents. [References].

Forster, Myriam; Grigsby, Timothy; Rogers, Christopher; Unger, Jennifer; Alvarado, Stephanie; Rainisch, Bethany; Areba, Eunice.

Journal of Adolescent Health. Vol.70(3), 2022, pp. 488-495.

[Journal; Peer Reviewed Journal]

Year of Publication

2022

Publication Month/Season

Mar

Cite

32.
The relations between the positive and negative components **of** self-compassion and **depressive symptoms** among sexual **minority** women and men. [References].

Shakeshaft, Rhianydd; McLaren, Suzanne.

Mindfulness. Vol.13(1), 2022, pp. 57-65.

[Journal; Peer Reviewed Journal]

Year of Publication

2022

Publication Month/Season

Jan

Cite

33.
Understanding unaccompanied immigrant youth's experiences **in** US schools: An interdisciplinary perspective. [References].

Frankel, Katherine K; Brabeck, Kalina M; Rendon Garcia, Sarah A.

Journal of Education for Students Placed at Risk. Vol.27(1), 2022, pp. 27-58.

[Journal; Peer Reviewed Journal]

Year of Publication

2022

Publication Month/Season

Jan-Mar

Cite

34.
Understanding the migration experience **of** unaccompanied **youth**: A review **of** the literature. [References].

Garcia, Maria Fernanda; Birman, Dina.

American Journal of Orthopsychiatry. Vol.92(1), 2022, pp. 79-102.

[Journal; Peer Reviewed Journal]

Year of Publication

2022

Cite

35.
Sexual orientation, social **support**, and **mental health** resilience **in** a U.S. national sample **of adults**. [References].

Krueger, Evan A; Upchurch, Dawn M.

Behavioral Medicine. Vol.48(3), 2022, pp. 207-215.

[Journal; Peer Reviewed Journal]

Year of Publication

2022

Publication Month/Season

Jul-Sep

Cite
