# Supplementary material for: Mental health and mental health help-seeking behaviors among first-generation voluntary African migrants: A systematic review
Source: PLoS One. 2024 Mar 18;19(3):e0298634. doi: 10.1371/journal.pone.0298634 (PMC10947684; doi:10.1371/journal.pone.0298634)
Supplement: S3 Appendix — A. CINAHL Search Strategy 16.12.2023. B. Embase Search Strategy 17.12.2023. C. Medline Complete Search Strategy 16.12.2023. D. PsychInfo Search Strategy 17.12.2023. (ZIP) [file pone.0298634.s005.zip › S3C_Appendix.pdf]

This XML file does not appear to have any style information associated with it. The document tree is shown below.

<records>
<rec resultID="1">
<header shortDbName="mnh" longDbName="MEDLINE with Full Text" uiTerm="32703193">
<controlInfo>
<bkinfo/>
<jinfo>
<jtl>BMC health services research</jtl>
<jtl>BMC Health Serv Res</jtl>
<issn type="Electronic">1472-6963</issn>
</jinfo>
<pubinfo>
<dt year="2020" month="07" day="23">2020 Jul 23</dt>
<vid>20</vid>
<iid>1</iid>
<place>England</place>
</pubinfo>
<artinfo>
<ui type="pmid">32703193</ui>
<ui type="doi">10.1186/s12913-020-05502-0</ui>
<pages>681</pages>
<formats>
<fmt type="HTML Full Text"/>
<fmt type="PDF"/>
</formats>
<tig>
<atl>Common mental disorders and its associated factors and mental health care services for Ethiopian labour migrants returned from Middle East countries in Addis Ababa, Ethiopia.</atl>
</tig>
<aug>
<au>Tilahun M</au>
<au>Workicho A</au>
<au>Angaw DA</au>
</aug>
<sug>
<subj type="geographic">Ethiopia</subj>
<subj type="geographic">Middle East</subj>
<subj type="major">Mental Disorders epidemiology</subj>
<subj type="major">Mental Disorders therapy</subj>
<subj type="major">Mental Health Services statistics & numerical data</subj>
<subj type="major">Transients and Migrants psychology</subj>
<subj type="unclass">Adult</subj>
<subj type="unclass">Ethiopia ethnology</subj>
<subj type="unclass">Female</subj>
<subj type="unclass">Humans</subj>
<subj type="unclass">Male</subj>
<subj type="unclass">Middle East epidemiology</subj>
<subj type="unclass">Risk Factors</subj>
<subj type="unclass">Transients and Migrants statistics & numerical data</subj>
<subj type="unclass">Young Adult</subj>
</sug>
<ab>The migration of young Ethiopian men and women to the Middle East countries was mainly for economic reasons. The migration was largely irregular that posed a wide range of unfavorable life conditions for some of the migrants. The overall objective is to assess common mental disorders and its associated factors for Ethiopian migrants returned from the Middle East countries and to describe mental health care services targeting these migrants.
</ab>
<ab>The study employed a mixed-methods approach. For the quantitative part, a systematic random sampling technique was used to select a sample of 517 returnees. An interviewer-administered questionnaire based on Self Report Questionnaire-20 was used to collect data from respondents. The qualitative study employed a phenomenological study design to

describe mental health care services. Key informant interviews and non-participant observation techniques were used to collect qualitative data.</ab>
<ab>The prevalence of common mental disorder among Ethiopian migrants returned from the Middle East countries was found to be 29.2%. education (AOR=2.90 95%CI: 1.21, 6.94), physical abuse (AOR=12.17 95%CI: 5.87, 25.22), not getting salary properly and timely (AOR=3.35 95%CI: 1.47, 7.63), history of mental illness in the family (AOR=6.75 95%CI: 1.03, 43.95), detention (AOR=4.74 95%CI: 2.60, 8.62), guilty feeling for not fulfilling goal (AOR=9.58 95%CI: 4.43, 20.71), and denial of access to health care (AOR=3.20 95%CI:1.53, 6.67) were significantly associated with a common mental disorder. Shelter based and hospital-based mental health care services were rendered for a few return migrants with mental disorders. The services were primarily targeted, female return migrants.</ab>
<ab>The prevalence of common mental disorder was high among migrants returned from the Middle East countries. Despite the high burden of mental distress, only a small proportion of return migrants with mental illness is getting mental health care services.</ab>
<pubtype>Journal Article</pubtype>
</artinfo>
<language code="eng">English</language>
</controlInfo>
<displayInfo>
<pLink>
<url>https://search.ebscohost.com/login.aspx?direct=true&AuthType=sso&db=mnh&AN=32703193&site=ehost-live&custid=s2775460</url>
</pLink>
</displayInfo>
</header>
</rec>
<rec resultID="2">
<header shortDbName="mnh" longDbName="MEDLINE with Full Text" uiTerm="33849300">
<controlInfo>
<bkinfo/>
<jinfo>
<jtl>Ethnicity & health</jtl>
<jtl>Ethn Health</jtl>
<issn type="Electronic">1465-3419</issn>
</jinfo>
<pubinfo>
<dt year="2022" month="10" day="01">2022 Oct</dt>
<vid>27</vid>
<iid>7</iid>
<place>England</place>
</pubinfo>
<artinfo>
<ui type="pmid">33849300</ui>
<ui type="doi">10.1080/13557858.2021.1910930</ui>
<pages>1501-1517</pages>
<formats/>
<tig>
<atl>Acculturative stress, stigma, and mental health challenges: emic perspectives from Somali young adults in San Diego county's 'Little Mogadishu'.</atl>
</tig>
<aug>
<au>Alemi Q</au>
<au>Mefom E</au>
<au>Montgomery S</au>
<au>Koga PM</au>
<au>Stempel C</au>
<au>Reimann JOF</au>
</aug>
<sug>
<subj type="geographic">Somalia</subj>
<subj type="major">Acculturation</subj>
<subj type="major">Mental Health Services</subj>
<subj type="unclass">Adolescent</subj>
<subj type="unclass">Child</subj>

```xml
          <subj type="unclass">Humans</subj>
          <subj type="unclass">Mental Health</subj>
          <subj type="unclass">Social Stigma</subj>
          <subj type="unclass">Somalia</subj>
          <subj type="unclass">Young Adult</subj>
        </sug>
        <ab>The purpose of this study was to explore the social factors that contribute to the
        mental health challenges that Somali young adults endure.</ab>
        <ab>In a two-phase qualitative approach carried-out in the San Diego area, in phase-I, we
        conducted exploratory interviews with key-informants including clinicians and local Somali
        leaders ( n  = 7) who are familiar with the challenges of young Somalis. This information
        was then augmented through a focus group discussion with Somali young adults ( n  = 4) to
        gain further contextual knowledge and for access to the larger community of young people
        for phase-II. In this second phase, we carried-out individual interviews with 21 Somali
        young adults. Interviews covered topics including the social factors influencing their
        mental health, typical strategies for coping with psychological distress, barriers to
        seeking professional mental health services, and suggestions for combating mental health
        problems affecting young Somalis.</ab>
        <ab>Participant narratives indicate that psychological distress (depression and
        posttraumatic stress disorder) are highly pervasive, and that shame, acculturative stress
        and ethnic discrimination as well as parents' dismissive reactions to their children's
        emotional problems perpetuate mental health problems. Coping strategies included support
        from friends, religious activities, and playing soccer. Suggestions for addressing their
        challenges centered on engagement from their own community to advocate for mental health.
        </ab>
        <ab>Implications of this study are discussed in the context of bridging intergenerational
        and acculturation divides to deliver culturally competent interventions that improve the
        mental health and well-being of Somali young adults and aid them in their adjustment to the
        U.S.</ab>
        <pubtype>Journal Article</pubtype>
      </artinfo>
      <language code="eng">English</language>
    </controlInfo>
    <displayInfo>
      <pLink>
        <url>https://search.ebscohost.com/login.aspx?
        direct=true&AuthType=sso&db=mnh&AN=33849300&site=ehost-live&custid=s2775460</url>
      </pLink>
    </displayInfo>
  </header>
</rec>
<rec resultID="3">
  <header shortDbName="mnh" longDbName="MEDLINE with Full Text" uiTerm="32409884">
    <controlInfo>
      <bkinfo/>
      <jinfo>
        <jtl>Social psychiatry and psychiatric epidemiology</jtl>
        <jtl>Soc Psychiatry Psychiatr Epidemiol</jtl>
        <issn type="Electronic">1433-9285</issn>
      </jinfo>
      <pubinfo>
        <dt year="2020" month="11" day="01">2020 Nov</dt>
        <vid>55</vid>
        <iid>11</iid>
        <place>Germany</place>
      </pubinfo>
      <artinfo>
        <ui type="pmid">32409884</ui>
        <ui type="doi">10.1007/s00127-020-01878-w</ui>
        <pages>1457-1468</pages>
        <formats>
          <fmt type="HTML Full Text"/>
          <fmt type="PDF"/>
        </formats>
        <tig>
```

<atl>Psychiatric healthcare utilisation among refugee adolescents and their peers in Denmark.</atl>
</tig>
<aug>
<au>de Montgomery CJ</au>
<au>Petersen JH</au>
<au>Jervelund SS</au>
</aug>
<sug>
<subj type="geographic">Denmark</subj>
<subj type="geographic">Morocco</subj>
<subj type="geographic">Pakistan</subj>
<subj type="geographic">Turkey</subj>
<subj type="major">Mental Health Services</subj>
<subj type="major">Refugees psychology</subj>
<subj type="unclass">Adolescent</subj>
<subj type="unclass">Adult</subj>
<subj type="unclass">Child</subj>
<subj type="unclass">Denmark epidemiology</subj>
<subj type="unclass">Ethnicity</subj>
<subj type="unclass">Female</subj>
<subj type="unclass">Humans</subj>
<subj type="unclass">Male</subj>
<subj type="unclass">Minority Groups</subj>
<subj type="unclass">Morocco</subj>
<subj type="unclass">Pakistan</subj>
<subj type="unclass">Turkey</subj>
<subj type="unclass">Young Adult</subj>
</sug>
<ab>To investigate the psychiatric healthcare utilisation of refugees vis-à-vis their peers in Denmark during the ages 15-22.</ab>
<ab>This paper utilises comprehensive full-population registry data from 1995 to 2016 to explore the psychiatric healthcare utilisation during the transition from childhood to adulthood for refugees (N = 13,027), a comparison group of children of labour migrants from Morocco, Pakistan, and Turkey (N = 13,413), and the majority population (N = 693,043) in Denmark. To test for population differences in types of admission for particular types of disorders, odds ratios for a first contact during ages 15-22 were calculated using logistic regression. For those with at least one diagnosis-specific hospital contact, differences in the amount and type of treatment were tested using negative binomial regression to estimate means ratios of days hospitalised, days in outpatient care, number of outpatient contacts, consultations with psychiatrists in private practice, and prescribed medicine purchases.
</ab>
<ab>Refugees and the comparison group were generally less likely than the majority population to have a first contact for most disorders (adjusted ORs 0.03-0.88), but not for schizophrenia for boys (adjusted ORs 0.92-2.13). Among those who did have a first contact, youths from the ethnic minority groups tended to have more or similar inpatient and emergency room contacts (MRs 0.89-2.10), hospitalisations of refugee girls being an exception (MR 0.46; CI [0.23-0.94]), but fewer outpatient contacts, consultations with psychiatrists in private practice, and prescribed medicine purchases (MRs 0.23-0.94).</ab>
<ab>The results suggest that refugee and other ethnic minority groups may face barriers both to initial contact and to completing adequate treatment beyond the first contact.</ab>
<pubtype>Journal Article</pubtype>
</artinfo>
<language code="eng">English</language>
</controlInfo>
<displayInfo>
<pLink>
<url>https://search.ebscohost.com/login.aspx?direct=true&AuthType=sso&db=mnh&AN=32409884&site=ehost-live&custid=s2775460</url>
</pLink>
</displayInfo>
</header>
</rec>
<rec resultID="4">
<header shortDbName="mnh" longDbName="MEDLINE with Full Text" uiTerm="32652988">

```xml
        <controlInfo>
          <bkinfo/>
          <jinfo>
            <jtl>BMC health services research</jtl>
            <jtl>BMC Health Serv Res</jtl>
            <issn type="Electronic">1472-6963</issn>
          </jinfo>
          <pubinfo>
            <dt year="2020" month="07" day="11">2020 Jul 11</dt>
            <vid>20</vid>
            <iid>1</iid>
            <place>England</place>
          </pubinfo>
          <artinfo>
            <ui type="pmid">32652988</ui>
            <ui type="doi">10.1186/s12913-020-05478-x</ui>
            <pages>648</pages>
            <formats>
              <fmt type="HTML Full Text"/>
              <fmt type="PDF"/>
            </formats>
            <tig>
              <atl>Immigration, acculturation, and preferred help-seeking sources for depression:
              comparison of five ethnic groups.</atl>
            </tig>
            <aug>
              <au>Markova V</au>
              <au>Sandal GM</au>
              <au>Pallesen S</au>
            </aug>
            <sug>
              <subj type="geographic">Norway</subj>
              <subj type="geographic">Pakistan</subj>
              <subj type="geographic">Poland</subj>
              <subj type="geographic">Russia</subj>
              <subj type="geographic">Somalia</subj>
              <subj type="major">Acculturation</subj>
              <subj type="major">Depression ethnology</subj>
              <subj type="major">Depression therapy</subj>
              <subj type="major">Emigrants and Immigrants psychology</subj>
              <subj type="major">Ethnicity psychology</subj>
              <subj type="major">Help-Seeking Behavior</subj>
              <subj type="major">Patient Preference statistics & numerical data</subj>
              <subj type="unclass">Adolescent</subj>
              <subj type="unclass">Adult</subj>
              <subj type="unclass">Emigrants and Immigrants statistics & numerical data</subj>
              <subj type="unclass">Ethnicity statistics & numerical data</subj>
              <subj type="unclass">Female</subj>
              <subj type="unclass">Humans</subj>
              <subj type="unclass">Male</subj>
              <subj type="unclass">Mental Health Services organization & administration</subj>
              <subj type="unclass">Mental Health Services statistics & numerical data</subj>
              <subj type="unclass">Norway ethnology</subj>
              <subj type="unclass">Pakistan ethnology</subj>
              <subj type="unclass">Poland ethnology</subj>
              <subj type="unclass">Russia ethnology</subj>
              <subj type="unclass">Somalia ethnology</subj>
              <subj type="unclass">Students psychology</subj>
              <subj type="unclass">Students statistics & numerical data</subj>
              <subj type="unclass">Surveys and Questionnaires</subj>
              <subj type="unclass">Young Adult</subj>
            </sug>
            <ab>Immigrants are more likely than the majority population to have unmet needs for public
            mental health services. This study aims to understand potential ethnic differences in
```

```
          preferred help-seeking sources for depression in Norway, and how such preferences relate to
          acculturation orientation.</ab>
          <ab>A convenience sample of immigrants from Russia (n = 164), Poland (n = 127), Pakistan
          (n = 128), and Somalia (n = 114), and Norwegian students (n = 250) completed a survey. The
          sample was recruited from social media platforms, emails, and direct contact. The survey
          consisted of a vignette describing a moderately depressed person. Respondents were asked to
          provide advice to the person by completing a modified version of the General Help-Seeking
          Questionnaire. The immigrant sample also responded to questions about acculturation
          orientation using the Vancouver Index of Acculturation Scale.</ab>
          <ab>Significant differences were found in the endorsement of traditional (e.g., religious
          leader), informal (e.g., family), and semiformal (e.g., internet forum) help-sources
          between immigrant groups, and between immigrant groups and the Norwegian respondent group.
          Immigrants from Pakistan and Somalia endorsed traditional help sources to a greater extent
          than immigrants from Russia and Poland, and the Norwegian student sample. There were no
          ethnic differences in endorsement of formal mental help sources (e.g., a medical doctor).
          Maintenance of the culture of origin as the acculturation orientation was associated with
          preferences for traditional and informal help sources, while the adoption of mainstream
          culture was associated with semiformal and formal help-seeking sources.</ab>
          <ab>Ethnic differences in help-seeking sources need to be considered when designing and
          implementing mental health services.</ab>
          <pubtype>Journal Article</pubtype>
          <doctype>Comparative Study</doctype>
        </artinfo>
        <language code="eng">English</language>
      </controlInfo>
      <displayInfo>
        <pLink>
          <url>https://search.ebscohost.com/login.aspx?
          direct=true&AuthType=sso&db=mnh&AN=32652988&site=ehost-live&custid=s2775460</url>
        </pLink>
      </displayInfo>
    </header>
  </rec>
  <rec resultID="5">
    <header shortDbName="mnh" longDbName="MEDLINE with Full Text" uiTerm="29441451">
      <controlInfo>
        <bkinfo/>
        <jinfo>
          <jtl>The journal of behavioral health services & research</jtl>
          <jtl>J Behav Health Serv Res</jtl>
          <issn type="Electronic">1556-3308</issn>
        </jinfo>
        <pubinfo>
          <dt year="2019" month="01" day="01">2019 Jan</dt>
          <vid>46</vid>
          <iid>1</iid>
          <place>United States</place>
        </pubinfo>
        <artinfo>
          <ui type="pmid">29441451</ui>
          <ui type="doi">10.1007/s11414-018-9587-x</ui>
          <pages>80-98</pages>
          <formats>
            <fmt type="HTML Full Text"/>
            <fmt type="PDF"/>
          </formats>
          <tig>
            <atl>Trauma, Psychosocial Factors, and Help-Seeking in Three Immigrant Groups in Finland.
            </atl>
          </tig>
          <aug>
            <au>Schubert CC</au>
            <au>Punamäki RL</au>
            <au>Suvisaari J</au>
            <au>Koponen P</au>
```

```xml
          <au>Castaneda A</au>
        </aug>
        <sug>
          <subj type="geographic">Finland</subj>
          <subj type="geographic">Russia</subj>
          <subj type="geographic">Somalia</subj>
          <subj type="major">Emigrants and Immigrants psychology</subj>
          <subj type="major">Emigrants and Immigrants statistics & numerical data</subj>
          <subj type="major">Help-Seeking Behavior</subj>
          <subj type="major">Mental Disorders psychology</subj>
          <subj type="major">Mental Health Services</subj>
          <subj type="unclass">Acculturation</subj>
          <subj type="unclass">Adolescent</subj>
          <subj type="unclass">Adult</subj>
          <subj type="unclass">Cross-Sectional Studies</subj>
          <subj type="unclass">Female</subj>
          <subj type="unclass">Finland</subj>
          <subj type="unclass">Humans</subj>
          <subj type="unclass">Interviews as Topic</subj>
          <subj type="unclass">Male</subj>
          <subj type="unclass">Mental Disorders therapy</subj>
          <subj type="unclass">Middle Aged</subj>
          <subj type="unclass">Russia ethnology</subj>
          <subj type="unclass">Somalia ethnology</subj>
          <subj type="unclass">Trauma and Stressor Related Disorders psychology</subj>
          <subj type="unclass">Trauma and Stressor Related Disorders therapy</subj>
          <subj type="unclass">Young Adult</subj>
        </sug>
        <ab>Multiple psychosocial factors influence help-seeking behavior among immigrants, but
        studies have focused on separate issues in single cultural groups. This study tested a
        model of help-seeking behavior among three ethnically different immigrant groups.
        Participants were 1356 Somali, Russian, and Kurdish immigrants (18-64 years). They reported
        past traumatic events, social network, acculturation indices, trust in services, and mental
        health as well as usage of mental and somatic health services. Structural equation modeling
        (SEM) with multigroup procedure was applied. First, past traumatic events were associated
        with seeking more mental health services, indirectly mediated through increased risk for
        mental health problems in all three ethnic groups. Second, acculturation played a
        significant role in the use of mental and somatic health services only in Kurds and social
        networks in Kurds and Russians. The unique culturally influenced dynamics in help-seeking
        behavior should be considered in the development of health services.</ab>
        <pubtype>Journal Article</pubtype>
      </artinfo>
      <language code="eng">English</language>
    </controlInfo>
    <displayInfo>
      <pLink>
        <url>https://search.ebscohost.com/login.aspx?
        direct=true&AuthType=sso&db=mnh&AN=29441451&site=ehost-live&custid=s2775460</url>
      </pLink>
    </displayInfo>
  </header>
</rec>
<rec resultID="6">
  <header shortDbName="mnh" longDbName="MEDLINE with Full Text" uiTerm="34974919">
    <controlInfo>
      <bkinfo/>
      <jinfo>
        <jtl>The Journal of adolescent health : official publication of the Society for Adolescent
        Medicine</jtl>
        <jtl>J Adolesc Health</jtl>
        <issn type="Electronic">1879-1972</issn>
      </jinfo>
      <pubinfo>
        <dt year="2022" month="03" day="01">2022 Mar</dt>
        <vid>70</vid>
```

<iid>3</iid>
<place>United States</place>
</pubinfo>
<artinfo>
<ui type="pmid">34974919</ui>
<ui type="doi">10.1016/j.jadohealth.2021.10.012</ui>
<pages>488-495</pages>
<formats/>
<tig>
<atl>Perceived Discrimination, Coping Styles, and Internalizing Symptoms Among a Community Sample of Hispanic and Somali Adolescents.</atl>
</tig>
<aug>
<au>Forster M</au>
<au>Grigsby T</au>
<au>Rogers C</au>
<au>Unger J</au>
<au>Alvarado S</au>
<au>Rainisch B</au>
<au>Areba E</au>
</aug>
<sug>
<subj type="geographic">Somalia</subj>
<subj type="major">Ethnicity</subj>
<subj type="major">Perceived Discrimination</subj>
<subj type="unclass">Adaptation, Psychological</subj>
<subj type="unclass">Adolescent</subj>
<subj type="unclass">Depression psychology</subj>
<subj type="unclass">Female</subj>
<subj type="unclass">Hispanic or Latino</subj>
<subj type="unclass">Humans</subj>
<subj type="unclass">Male</subj>
<subj type="unclass">Minority Groups psychology</subj>
<subj type="unclass">Somalia</subj>
</sug>
<ab>Perceived discrimination, perceptions of receiving differential treatment due to negative attitudes, and stereotypes about one's racial/ethnic group can increase vulnerability to depression and anxiety. Although ethnic minority youth now represent over half of the U.S. youth population, few studies have investigated potential protective factors in the relationship between perceived discrimination and mental health across diverse ethnic minority immigrant youth from different cultural backgrounds.</ab>
<ab>We examined the association between perceived discrimination and past week symptoms of depression and anxiety and whether patterns of problem and emotion-focused coping moderate these relationships among Somali and Hispanic immigrant youth (N = 353) in an urban midwestern setting (mean age = 15; 53% male, 39% first generation, 75% low income). Path analysis models examined the main effects of perceived discrimination for depression and anxiety and whether problem and emotion-focused coping moderated these associations.</ab>
<ab>Path analysis models suggest that perceived discrimination was positively associated with past week symptoms of depression (β = .37, standard error = .06) and anxiety (β = .16, standard error = .06) across ethnicity. However, adolescents who reported high levels of discrimination and who used predominantly problem-focused coping strategies experienced fewer internalizing problems than youth who relied predominantly on emotion-focused coping strategies.</ab>
<ab>Our findings suggest that strengthening youths' problem-focused coping strategies in the face of discriminatory stress is a promising health promotion and risk prevention approach.</ab>
<pubtype>Journal Article</pubtype>
<doctype>Research Support, Non-U.S. Gov't</doctype>
</artinfo>
<language code="eng">English</language>
</controlInfo>
<displayInfo>
<pLink>
<url>https://search.ebscohost.com/login.aspx?direct=true&AuthType=sso&db=mnh&AN=34974919&site=ehost-live&custid=s2775460</url>

```
        </pLink>
      </displayInfo>
    </header>
  </rec>
  <rec resultID="7">
    <header shortDbName="mnh" longDbName="MEDLINE with Full Text" uiTerm="33715289">
      <controlInfo>
        <bkinfo/>
        <jinfo>
          <jtl>International journal of mental health nursing</jtl>
          <jtl>Int J Ment Health Nurs</jtl>
          <issn type="Electronic">1447-0349</issn>
        </jinfo>
        <pubinfo>
          <dt year="2021" month="08" day="01">2021 Aug</dt>
          <vid>30</vid>
          <iid>4</iid>
          <place>Australia</place>
        </pubinfo>
        <artinfo>
          <ui type="pmid">33715289</ui>
          <ui type="doi">10.1111/inm.12846</ui>
          <pages>931-938</pages>
          <formats/>
          <tig>
            <atl>Barriers to accessing mental health services in Somali-Australian women: a
            qualitative study.</atl>
          </tig>
          <aug>
            <au>Said M</au>
            <au>Boardman G</au>
            <au>Kidd S</au>
          </aug>
          <sug>
            <subj type="geographic">Australia</subj>
            <subj type="geographic">Somalia</subj>
            <subj type="major">Mental Health Services</subj>
            <subj type="unclass">Australia epidemiology</subj>
            <subj type="unclass">Female</subj>
            <subj type="unclass">Humans</subj>
            <subj type="unclass">Qualitative Research</subj>
            <subj type="unclass">Social Stigma</subj>
            <subj type="unclass">Somalia</subj>
          </sug>
          <ab>Despite the global prevalence of mental disorders being widely acknowledged, mental
          illness, complex trauma and the significant impact on individuals, families and communities
          continues to be poorly recognized, under-diagnosed and underreported. Based on the 2017
          Australian census, one-in-five (20%) people have experienced some type of mental illness
          within the last 12 months (Australian Bureau of Statistics [ABS], 2019). The prevalence
          rate of mental illness in culturally and linguistically diverse (CALD) communities is
          difficult to estimate due to cultural and linguistic issues and underutilization of mental
          health services. In particular, little epidemiological data is available about the
          prevalence of mental illness in the Somali-Australian community. The aim of this study was
          to identify the perceived barriers to help-seeking for mental health for Somali-Australian
          women. A qualitative descriptive study incorporating focus group discussions with 31
          Somali-Australian women was conducted in Melbourne, Australia. Braun & Clarke's (2006)
          thematic analysis was applied to the data. Four themes relating to help-seeking barriers
          were abstracted. Influence of faith explored how Islam can impact the person views on
          mental illness. Stigma focused on the relationship between public and self-stigma and help-
          seeking. Mistrust of Western healthcare system describes the participants concerns about
          the cultural disconnect between the community and the Western healthcare system. Finally,
          denial of mental illness reflected the community views on mental health. This study
          provides an insight into the factors that influence the Somali-Australian community help-
          seeking with mental health services. The findings have implications for mental health
          professionals and the Somali-Australian community.</ab>
```

<pubtype>Journal Article</pubtype>
</artinfo>
<language code="eng">English</language>
</controlInfo>
<displayInfo>
<pLink>
<url>https://search.ebscohost.com/login.aspx?direct=true&AuthType=sso&db=mnh&AN=33715289&site=ehost-live&custid=s2775460</url>
</pLink>
</displayInfo>
</header>
</rec>
<rec resultID="8">
<header shortDbName="mnh" longDbName="MEDLINE with Full Text" uiTerm="32370772">
<controlInfo>
<bkinfo/>
<jinfo>
<jtl>Health and quality of life outcomes</jtl>
<jtl>Health Qual Life Outcomes</jtl>
<issn type="Electronic">1477-7525</issn>
</jinfo>
<pubinfo>
<dt year="2020" month="05" day="05">2020 May 05</dt>
<vid>18</vid>
<iid>1</iid>
<place>England</place>
</pubinfo>
<artinfo>
<ui type="pmid">32370772</ui>
<ui type="doi">10.1186/s12955-020-01352-w</ui>
<pages>120</pages>
<formats>
<fmt type="HTML Full Text"/>
<fmt type="PDF"/>
</formats>
<tig>
<atl>Informing the measurement of wellbeing among young people living with HIV in sub-Saharan Africa for policy evaluations: a mixed-methods systematic review.</atl>
</tig>
<aug>
<au>Govindasamy D</au>
<au>Seeley J</au>
<au>Olaru ID</au>
<au>Wiyeh A</au>
<au>Mathews C</au>
<au>Ferrari G</au>
</aug>
<sug>
<subj type="geographic">Africa South of the Sahara</subj>
<subj type="major">HIV Infections psychology</subj>
<subj type="major">Quality of Life</subj>
<subj type="unclass">Adaptation, Psychological</subj>
<subj type="unclass">Adolescent</subj>
<subj type="unclass">Adult</subj>
<subj type="unclass">Africa South of the Sahara</subj>
<subj type="unclass">Female</subj>
<subj type="unclass">Humans</subj>
<subj type="unclass">Male</subj>
<subj type="unclass">Qualitative Research</subj>
<subj type="unclass">Social Stigma</subj>
<subj type="unclass">Social Support</subj>
<subj type="unclass">Young Adult</subj>
</sug>
<ab>Young people living with HIV (YPLHIV) in sub-Saharan Africa (SSA) are at high risk of having a poor quality of life. Addressing wellbeing explicitly within HIV/AIDS policies

could assist mitigation efforts. However, guidance on wellbeing measures to evaluate policies for YPLHIV is scarce. The aims of this mixed-methods review were to identify: i) key dimensions of wellbeing and ii) wellbeing measures that align to these dimensions among YPLHIV (15-24 years) in SSA. We searched six social science and medical databases, including grey literature. We included studies that examined correlates and lived experiences of wellbeing, among YPLHIV in SSA, from January 2000 to May 2019. Two reviewers independently screened abstracts and full texts and assessed methodological quality of included articles. We analysed quantitative and qualitative data using descriptive and meta-ethnographic approaches, respectively. Thereafter, we integrated findings using a framework approach. We identified 6527 citations. Of these, 10 quantitative and 30 qualitative studies were included. Being male, higher educational status, less stigma and more social support were likely correlates of wellbeing. Themes that shaped experiences suggestive of wellbeing were: 1) acceptance and belonging- stigma, social support; 2) coping; 3) standard of living. Our final synthesis found that the following dimensions potentially characterise wellbeing: self-acceptance, belonging, autonomy; positive relations, environmental mastery, purpose in life. Wellbeing for YPLHIV is multi-dimensional and relational. Relevant measures include the Personal Wellbeing Index, Ryff's Psychological Wellbeing Scale and Mental Health Continuum Short Form. However, psychometric evaluations of these scales among YPLHIV in SSA are needed.</ab>
<pubtype>Journal Article</pubtype>
<doctype>Systematic Review</doctype>
</artinfo>
<language code="eng">English</language>
</controlInfo>
<displayInfo>
<pLink>
<url>https://search.ebscohost.com/login.aspx?direct=true&AuthType=sso&db=mnh&AN=32370772&site=ehost-live&custid=s2775460</url>
</pLink>
</displayInfo>
</header>
</rec>
<rec resultID="9">
<header shortDbName="mnh" longDbName="MEDLINE with Full Text" uiTerm="32819364">
<controlInfo>
<bkinfo/>
<jinfo>
<jtl>International journal for equity in health</jtl>
<jtl>Int J Equity Health</jtl>
<issn type="Electronic">1475-9276</issn>
</jinfo>
<pubinfo>
<dt year="2020" month="08" day="20">2020 Aug 20</dt>
<vid>19</vid>
<iid>1</iid>
<place>England</place>
</pubinfo>
<artinfo>
<ui type="pmid">32819364</ui>
<ui type="doi">10.1186/s12939-020-01259-4</ui>
<pages>141</pages>
<formats/>
<tig>
<atl>Unspoken inequality: how COVID-19 has exacerbated existing vulnerabilities of asylum-seekers, refugees, and undocumented migrants in South Africa.</atl>
</tig>
<aug>
<au>Mukumbang FC</au>
<au>Ambe AN</au>
<au>Adebiyi BO</au>
</aug>
<sug>
<subj type="geographic">South Africa</subj>
<subj type="major">Coronavirus Infections epidemiology</subj>
<subj type="major">Health Status Disparities</subj>

<subj type="major">Pneumonia, Viral epidemiology</subj>
<subj type="major">Refugees statistics & numerical data</subj>
<subj type="major">Undocumented Immigrants statistics & numerical data</subj>
<subj type="major">Vulnerable Populations statistics & numerical data</subj>
<subj type="unclass">Adolescent</subj>
<subj type="unclass">Adult</subj>
<subj type="unclass">COVID-19</subj>
<subj type="unclass">Coronavirus Infections prevention & control</subj>
<subj type="unclass">Government</subj>
<subj type="unclass">Humans</subj>
<subj type="unclass">Middle Aged</subj>
<subj type="unclass">Pandemics prevention & control</subj>
<subj type="unclass">Pneumonia, Viral prevention & control</subj>
<subj type="unclass">Socioeconomic Factors</subj>
<subj type="unclass">South Africa epidemiology</subj>
<subj type="unclass">Young Adult</subj>
</sug>
<ab>An estimated 2 million foreign-born migrants of working age (15-64) were living in South Africa (SA) in 2017. Structural and practical xenophobia has driven asylum-seekers, refugees, and undocumented migrants in SA to abject poverty and misery. The Coronavirus Disease 2019 (COVID-19) containment measures adopted by the SA government through the lockdown of the nation have tremendously deepened the unequal treatment of asylum-seekers and refugees in SA. This can be seen through the South African government's lack of consideration of this marginalized population in economic, poverty, and hunger alleviation schemes. Leaving this category of our society out of the national response safety nets may lead to negative coping strategies causing mental health issues and secondary health concerns. An effective response to the socioeconomic challenges imposed by the COVID-19 pandemic should consider the economic and health impact of the pandemic on asylum-seekers, refugees, and undocumented migrants.</ab>
<pubtype>Commentary</pubtype>
<doctype>Letter</doctype>
</artinfo>
<language code="eng">English</language>
</controlInfo>
<displayInfo>
<pLink>
<url>https://search.ebscohost.com/login.aspx?direct=true&AuthType=sso&db=mnh&AN=32819364&site=ehost-live&custid=s2775460</url>
</pLink>
</displayInfo>
</header>
</rec>
<rec resultID="10">
<header shortDbName="mnh" longDbName="MEDLINE with Full Text" uiTerm="34825128">
<controlInfo>
<bkinfo/>
<jinfo>
<jtl>General psychiatry</jtl>
<jtl>Gen Psychiatr</jtl>
<issn type="Print">2517-729X</issn>
</jinfo>
<pubinfo>
<dt year="2021" month="11" day="09">2021 Nov 09</dt>
<vid>34</vid>
<iid>6</iid>
<place>England</place>
</pubinfo>
<artinfo>
<ui type="pmid">34825128</ui>
<ui type="doi">10.1136/gpsych-2021-100648</ui>
<pages>e100648</pages>
<formats/>
<tig>
<atl>Are climate change and mental health correlated?</atl>
</tig>

```
        <aug>
          <au>Ramadan AMH</au>
          <au>Ataallah AG</au>
        </aug>
        <sug/>
        <ab>Climate change is one of the biggest challenges of our time and is likely to affect
        human beings in substantial ways. Recently, researchers started paying more attention to
        the changes in climate and their subsequent impact on the social, environmental and
        economic determinants of health, and the role they play in causing or exacerbating mental
        health problems. The effects of climate change-related events on mental well-being could be
        classified into direct and indirect effects. The direct effects of climate change mostly
        occur after acute weather events and include post-traumatic stress disorder, anxiety,
        substance abuse disorder, depression and even suicidal ideation. The indirect effects
        include economic losses, displacement and forced migration, competition over scarce
        resources and collective violence. The risk factors for developing those mental health
        issues include young age, female gender, low socioeconomic status, loss or injury of a
        loved one, being a member of immigrant groups or indigenous people, pre-existing mental
        illness and inadequate social support. However, in some individuals, especially those
        undisturbed by any directly observable effects of climate change, abstract awareness and
        acknowledgement of the ongoing climate crisis can induce negative emotions that can be
        intense enough to cause mental health illness. Coping strategies should be provided to the
        affected communities to protect their mental health from collapse in the face of climate
        disasters. Awareness of the mental health impacts of climate change should be raised,
        especially in the high-risk groups. Social and global attention to the climate crisis and
        its detrimental effects on mental health are crucial. This paper was written with the aim
        of trying to understand the currently, scientifically proven impact of climate change-
        related disasters on mental health and understanding the different methods of solving the
        problem at the corporate level, by trying to decrease greenhouse gas emissions to zero, and
        at the individual level by learning how to cope with the impacts of those disasters.</ab>
        <ab>Competing interests: None declared.</ab>
        <pubtype>Journal Article</pubtype>
        <doctype>Review</doctype>
      </artinfo>
      <language code="eng">English</language>
    </controlInfo>
    <displayInfo>
      <pLink>
        <url>https://search.ebscohost.com/login.aspx?
        direct=true&AuthType=sso&db=mnh&AN=34825128&site=ehost-live&custid=s2775460</url>
      </pLink>
    </displayInfo>
  </header>
</rec>
<rec resultID="11">
  <header shortDbName="mnh" longDbName="MEDLINE with Full Text" uiTerm="28378064">
    <controlInfo>
      <bkinfo/>
      <jinfo>
        <jtl>Social psychiatry and psychiatric epidemiology</jtl>
        <jtl>Soc Psychiatry Psychiatr Epidemiol</jtl>
        <issn type="Electronic">1433-9285</issn>
      </jinfo>
      <pubinfo>
        <dt year="2017" month="06" day="01">2017 Jun</dt>
        <vid>52</vid>
        <iid>6</iid>
        <place>Germany</place>
      </pubinfo>
      <artinfo>
        <ui type="pmid">28378064</ui>
        <ui type="doi">10.1007/s00127-017-1381-1</ui>
        <pages>679-687</pages>
        <formats>
          <fmt type="PDF"/>
        </formats>
```

<tig>
<atl>Immigrants' utilization of specialist mental healthcare according to age, country of origin, and migration history: a nation-wide register study in Norway.</atl>
</tig>
<aug>
<au>Abebe DS</au>
<au>Lien L</au>
<au>Elstad JI</au>
</aug>
<sug>
<subj type="geographic">Iran</subj>
<subj type="geographic">Iraq</subj>
<subj type="geographic">Norway</subj>
<subj type="geographic">Poland</subj>
<subj type="geographic">Somalia</subj>
<subj type="geographic">Vietnam</subj>
<subj type="major">Emigrants and Immigrants statistics & numerical data</subj>
<subj type="major">Ethnicity statistics & numerical data</subj>
<subj type="major">Mental Health Services statistics & numerical data</subj>
<subj type="major">Patient Acceptance of Health Care statistics & numerical data</subj>
<subj type="major">Refugees statistics & numerical data</subj>
<subj type="unclass">Adolescent</subj>
<subj type="unclass">Adult</subj>
<subj type="unclass">Child</subj>
<subj type="unclass">Child, Preschool</subj>
<subj type="unclass">Emigration and Immigration</subj>
<subj type="unclass">Female</subj>
<subj type="unclass">Humans</subj>
<subj type="unclass">Infant</subj>
<subj type="unclass">Infant, Newborn</subj>
<subj type="unclass">Iran ethnology</subj>
<subj type="unclass">Iraq ethnology</subj>
<subj type="unclass">Male</subj>
<subj type="unclass">Middle Aged</subj>
<subj type="unclass">Norway</subj>
<subj type="unclass">Patient Acceptance of Health Care ethnology</subj>
<subj type="unclass">Poland ethnology</subj>
<subj type="unclass">Registries</subj>
<subj type="unclass">Somalia ethnology</subj>
<subj type="unclass">Vietnam ethnology</subj>
<subj type="unclass">Young Adult</subj>
</sug>
<ab>As the immigrant population rises in Norway, it becomes ever more important to consider the responsiveness of health services to the specific needs of these immigrants. It has been questioned whether access to mental healthcare is adequate among all groups of immigrants. This study aims to examine the use of specialist mental healthcare services among ethnic Norwegians and specific immigrants groups.</ab>
<ab>Register data were used from the Norwegian Patient Registry and Statistics Norway. The sample (age 0-59) consisted of 3.3 million ethnic Norwegians and 200,000 immigrants from 11 countries. Poisson regression models were applied to examine variations in the use of specialist mental healthcare during 2008-2011 according to country of origin, age group, reason for immigration, and length of stay.</ab>
<ab>Immigrant children and adolescents had overall significantly lower use of specialist mental healthcare than ethnic Norwegians of the same age. A distinct exception was the high utilization rate among children and youth from Iran. Among adult immigrants, utilization rates were generally lower than among ethnic Norwegians, particularly those from Poland, Somalia, Sri Lanka, and Vietnam. Adult immigrants from Iraq and Iran, however, had high utilization rates. Refugees had high utilization rates of specialist mental healthcare, while labour immigrants had low use.</ab>
<ab>Utilization rates of specialist mental healthcare are lower among immigrants than Norwegians. Immigrants from Poland, Somalia, Sri Lanka, and Vietnam, had generally quite low rates, while immigrants from Iran had high utilization rates. The findings suggest that specialist mental healthcare in Norway is underutilized among considerable parts of the immigrant population.</ab>
<pubtype>Journal Article</pubtype>

</artinfo>
<language code="eng">English</language>
</controlInfo>
<displayInfo>
<pLink>
<url>https://search.ebscohost.com/login.aspx?direct=true&AuthType=sso&db=mnh&AN=28378064&site=ehost-live&custid=s2775460</url>
</pLink>
</displayInfo>
</header>
</rec>
<rec resultID="12">
<header shortDbName="mnh" longDbName="MEDLINE with Full Text" uiTerm="31605127">
<controlInfo>
<bkinfo/>
<jinfo>
<jtl>The Gerontologist</jtl>
<jtl>Gerontologist</jtl>
<issn type="Electronic">1758-5341</issn>
</jinfo>
<pubinfo>
<dt year="2020" month="02" day="24">2020 Feb 24</dt>
<vid>60</vid>
<iid>2</iid>
<place>United States</place>
</pubinfo>
<artinfo>
<ui type="pmid">31605127</ui>
<ui type="doi">10.1093/geront/gnz129</ui>
<pages>259-269</pages>
<formats/>
<tig>
<atl>Psychological Resilience in the Context of Disability: A Study With Turkish and Moroccan Young-Old Immigrants Living in the Netherlands.</atl>
</tig>
<aug>
<au>Szabó Á</au>
<au>Klokgieters SS</au>
<au>Kok AAL</au>
<au>van Tilburg TG</au>
<au>Huisman M</au>
</aug>
<sug>
<subj type="geographic">Morocco</subj>
<subj type="geographic">Netherlands</subj>
<subj type="geographic">Turkey</subj>
<subj type="major">Adaptation, Psychological</subj>
<subj type="major">Disabled Persons psychology</subj>
<subj type="major">Emigrants and Immigrants psychology</subj>
<subj type="major">Resilience, Psychological</subj>
<subj type="major">Transients and Migrants psychology</subj>
<subj type="unclass">Aged</subj>
<subj type="unclass">Female</subj>
<subj type="unclass">Humans</subj>
<subj type="unclass">Male</subj>
<subj type="unclass">Mental Health ethnology</subj>
<subj type="unclass">Middle Aged</subj>
<subj type="unclass">Morocco</subj>
<subj type="unclass">Netherlands ethnology</subj>
<subj type="unclass">Quality of Life</subj>
<subj type="unclass">Social Support</subj>
<subj type="unclass">Socioeconomic Factors</subj>
<subj type="unclass">Turkey</subj>
</sug>

```xml
        <ab>The disability paradox postulates that some individuals with severe functional
        limitations demonstrate psychological resilience, that is, good mental health and quality
        of life. Resilience to disabilities has been linked to psychological (e.g., mastery) and
        social factors (e.g., social provisions). It is, however, less clear whether cultural
        factors can provide additional resources for resilience building in older immigrants. We
        investigated the extent to which sociodemographic, psychosocial, and cultural factors
        contributed to psychological resilience to disabilities among immigrants of Turkish and
        Moroccan descent in the Netherlands.</ab>
        <ab>The sample included 478 older immigrants aged 55-65 years. Data were analyzed using
        latent profile analysis and multinomial logistic regressions.</ab>
        <ab>Five categories were identified: (a) High physical and emotional functioning; (b) High
        physical but poor emotional functioning; (c) Low physical but high emotional functioning
        (resilient); (d) Low physical and emotional functioning; and (e) Low physical and very low
        emotional functioning. Resilient functioning (reference category) was associated with
        poorer Dutch language proficiency, lower levels of loneliness, greater mastery, and more
        religious coping.</ab>
        <ab>Findings provide support for the disability paradox and highlight social provisions,
        mastery, and religiosity/spirituality as important resources for psychological resilience
        in older labor migrants. Poor Dutch language proficiency is discussed as a potential factor
        contributing to severe functional limitations in the resilient category.</ab>
        <pubtype>Journal Article</pubtype>
        <doctype>Research Support, Non-U.S. Gov't</doctype>
      </artinfo>
      <language code="eng">English</language>
    </controlInfo>
    <displayInfo>
      <pLink>
        <url>https://search.ebscohost.com/login.aspx?
        direct=true&AuthType=sso&db=mnh&AN=31605127&site=ehost-live&custid=s2775460</url>
      </pLink>
    </displayInfo>
  </header>
</rec>
<rec resultID="13">
  <header shortDbName="mnh" longDbName="MEDLINE with Full Text" uiTerm="24912873">
    <controlInfo>
      <bkinfo/>
      <jinfo>
        <jtl>American journal of community psychology</jtl>
        <jtl>Am J Community Psychol</jtl>
        <issn type="Electronic">1573-2770</issn>
      </jinfo>
      <pubinfo>
        <dt year="2014" month="09" day="01">2014 Sep</dt>
        <vid>54</vid>
        <iid>1-2</iid>
        <place>England</place>
      </pubinfo>
      <artinfo>
        <ui type="pmid">24912873</ui>
        <ui type="doi">10.1007/s10464-014-9663-1</ui>
        <pages>1-11</pages>
        <formats>
          <fmt type="HTML Full Text"/>
          <fmt type="PDF"/>
        </formats>
        <tig>
          <atl>Well-being and social justice among Moroccan migrants in southern Spain.</atl>
        </tig>
        <aug>
          <au>Paloma V</au>
          <au>García-Ramírez M</au>
          <au>Camacho C</au>
        </aug>
        <sug>
```

```xml
          <subj type="geographic">Morocco</subj>
          <subj type="geographic">Spain</subj>
          <subj type="major">Emigrants and Immigrants psychology</subj>
          <subj type="major">Mental Health</subj>
          <subj type="major">Personal Satisfaction</subj>
          <subj type="major">Social Justice psychology</subj>
          <subj type="major">Stress, Psychological psychology</subj>
          <subj type="unclass">Adaptation, Psychological</subj>
          <subj type="unclass">Adult</subj>
          <subj type="unclass">Female</subj>
          <subj type="unclass">Humans</subj>
          <subj type="unclass">Male</subj>
          <subj type="unclass">Models, Psychological</subj>
          <subj type="unclass">Morocco ethnology</subj>
          <subj type="unclass">Multilevel Analysis</subj>
          <subj type="unclass">Prejudice psychology</subj>
          <subj type="unclass">Regression Analysis</subj>
          <subj type="unclass">Social Marginalization psychology</subj>
          <subj type="unclass">Spain</subj>
          <subj type="unclass">Young Adult</subj>
        </sug>
        <ab>The decision to migrate is normally based on expectations of improving one's actual
        living conditions and therefore, one's well-being. However, these expectations are not
        usually met in receiving contexts that relegate newcomers to lower power positions. From a
        liberating community psychology approach, this study aims to develop a predictive model of
        the well-being of Moroccan migrants living in southern Spain. Data were collected from a
        survey sample of 633 migrants (the average age was 31.9 years and 51.8 % were women) from
        20 territorial units of Andalusia. Through a process of multilevel regression analysis,
        this study reveals that the well-being of the Moroccan community is closely determined by
        the following: (a) the level of social justice in the receiving context (openness to
        diversity of receiving communities, cultural sensitivity of community services, and
        residential integration); and (b) the individual strengths of the population (use of active
        coping strategies, satisfaction with the receiving context, and temporal stability in the
        new environment). These results empirically support the impact that different ecological
        levels of analysis have on well-being. Major theoretical contributions of the model and
        useful suggestions for improving migrant well-being are discussed.</ab>
        <pubtype>Journal Article</pubtype>
        <doctype>Research Support, Non-U.S. Gov't</doctype>
      </artinfo>
      <language code="eng">English</language>
    </controlInfo>
    <displayInfo>
      <pLink>
        <url>https://search.ebscohost.com/login.aspx?
        direct=true&AuthType=sso&db=mnh&AN=24912873&site=ehost-live&custid=s2775460</url>
      </pLink>
    </displayInfo>
  </header>
</rec>
<rec resultID="14">
  <header shortDbName="mnh" longDbName="MEDLINE with Full Text" uiTerm="24976522">
    <controlInfo>
      <bkinfo/>
      <jinfo>
        <jtl>Social psychiatry and psychiatric epidemiology</jtl>
        <jtl>Soc Psychiatry Psychiatr Epidemiol</jtl>
        <issn type="Electronic">1433-9285</issn>
      </jinfo>
      <pubinfo>
        <dt year="2015" month="01" day="01">2015 Jan</dt>
        <vid>50</vid>
        <iid>1</iid>
        <place>Germany</place>
      </pubinfo>
      <artinfo>
```

```
<ui type="pmid">24976522</ui>
<ui type="doi">10.1007/s00127-014-0916-y</ui>
<pages>67-76</pages>
<formats>
  <fmt type="PDF"/>
</formats>
<tig>
  <atl>Utilisation of psychiatrists and psychologists in private practice among non-Western
  labour immigrants, immigrants from refugee-generating countries and ethnic Danes: the
  role of mental health status.</atl>
</tig>
<aug>
  <au>Nielsen SS</au>
  <au>Jensen NK</au>
  <au>Kreiner S</au>
  <au>Norredam M</au>
  <au>Krasnik A</au>
</aug>
<sug>
  <subj type="geographic">Denmark</subj>
  <subj type="geographic">Iran</subj>
  <subj type="geographic">Iraq</subj>
  <subj type="geographic">Lebanon</subj>
  <subj type="geographic">Pakistan</subj>
  <subj type="geographic">Somalia</subj>
  <subj type="geographic">Turkey</subj>
  <subj type="major">Ambulatory Care statistics & numerical data</subj>
  <subj type="major">Emigrants and Immigrants statistics & numerical data</subj>
  <subj type="major">Health Services Needs and Demand statistics & numerical data</subj>
  <subj type="major">Mental Health Services statistics & numerical data</subj>
  <subj type="major">Private Practice statistics & numerical data</subj>
  <subj type="unclass">Adolescent</subj>
  <subj type="unclass">Adult</subj>
  <subj type="unclass">Aged</subj>
  <subj type="unclass">Denmark epidemiology</subj>
  <subj type="unclass">Ethnicity classification</subj>
  <subj type="unclass">Ethnicity statistics & numerical data</subj>
  <subj type="unclass">Female</subj>
  <subj type="unclass">Health Status</subj>
  <subj type="unclass">Health Surveys</subj>
  <subj type="unclass">Humans</subj>
  <subj type="unclass">Iran ethnology</subj>
  <subj type="unclass">Iraq ethnology</subj>
  <subj type="unclass">Lebanon ethnology</subj>
  <subj type="unclass">Male</subj>
  <subj type="unclass">Middle Aged</subj>
  <subj type="unclass">Pakistan ethnology</subj>
  <subj type="unclass">Population Groups</subj>
  <subj type="unclass">Psychiatry statistics & numerical data</subj>
  <subj type="unclass">Psychology statistics & numerical data</subj>
  <subj type="unclass">Refugees statistics & numerical data</subj>
  <subj type="unclass">Registries statistics & numerical data</subj>
  <subj type="unclass">Sex Factors</subj>
  <subj type="unclass">Socioeconomic Factors</subj>
  <subj type="unclass">Somalia ethnology</subj>
  <subj type="unclass">Turkey ethnology</subj>
  <subj type="unclass">Young Adult</subj>
</sug>
<ab>The stressful migration process has been associated with higher vulnerability for
mental health problems, implying a greater need for mental healthcare among immigrants
compared with native-born. Our objective was to investigate whether potential differences
in the use of psychiatrists and psychologists in labour immigrants, immigrants from
refugee-generating countries (RGC), and ethnic Danes could be fully explained by mental
health status.</ab>
```

<ab>We conducted a nationwide survey in 2007 with 3,573 individuals aged 18-66 comprising ethnic Danes, labour immigrants (Pakistan and Turkey), and immigrants from RGC (Iran, Iraq, Lebanon, and Somalia). Survey data was linked to healthcare utilisation registries. Using Poisson regression, contacts with private practising psychiatrists and psychologists were estimated. Analyses were adjusted for socioeconomic factors and mental health status.</ab>
<ab>Overall, 2.2 % among ethnic Danes, 1.4 % among labour immigrants and 6.5 % among immigrants from RGC consulted a psychiatrist or psychologist. In adjusted analyses, for psychiatrists, compared with ethnic Danes, labour-immigrant women (multiplicative effect = 1.78), and immigrant women from RGC (multiplicative effect = 2.49) had increased use, while labour-immigrant men had decreased use (multiplicative effect = 0.03). For psychologists, immigrant men from RGC had increased use (multiplicative effect = 2.96), while labour-immigrant women had decreased use (multiplicative effect = 0.27) compared with ethnic Danes.</ab>
<ab>Mental health status had a somewhat explanatory effect on the use of psychiatrists and psychologists. These selected parts of the Danish mental healthcare system seem responsive to health needs across different population groups, particularly for immigrants from RGC. Yet more attention should be given to non-Western labour immigrants to meet their mental health needs.</ab>
<pubtype>Journal Article</pubtype>
<doctype>Research Support, Non-U.S. Gov't</doctype>
</artinfo>
<language code="eng">English</language>
</controlInfo>
<displayInfo>
<pLink>
<url>https://search.ebscohost.com/login.aspx?direct=true&AuthType=sso&db=mnh&AN=24976522&site=ehost-live&custid=s2775460</url>
</pLink>
</displayInfo>
</header>
</rec>
<rec resultID="15">
<header shortDbName="mnh" longDbName="MEDLINE with Full Text" uiTerm="30222038">
<controlInfo>
<bkinfo/>
<jinfo>
<jtl>Qualitative health research</jtl>
<jtl>Qual Health Res</jtl>
<issn type="Print">1049-7323</issn>
</jinfo>
<pubinfo>
<dt year="2019" month="01" day="01">2019 Jan</dt>
<vid>29</vid>
<iid>2</iid>
<place>United States</place>
</pubinfo>
<artinfo>
<ui type="pmid">30222038</ui>
<ui type="doi">10.1177/1049732318800004</ui>
<pages>222-236</pages>
<formats/>
<tig>
<atl>Social Resilience and Mental Health Among Eritrean Asylum-Seekers in Switzerland.
</atl>
</tig>
<aug>
<au>Melamed S</au>
<au>Chernet A</au>
<au>Labhardt ND</au>
<au>Probst-Hensch N</au>
<au>Pfeiffer C</au>
</aug>
<sug>
<subj type="geographic">Eritrea</subj>
<subj type="geographic">Switzerland</subj>

<subj type="major">Emigrants and Immigrants psychology</subj>
<subj type="major">Mental Health ethnology</subj>
<subj type="major">Mental Health Services organization & administration</subj>
<subj type="major">Refugees psychology</subj>
<subj type="major">Resilience, Psychological</subj>
<subj type="unclass">Adult</subj>
<subj type="unclass">Eritrea ethnology</subj>
<subj type="unclass">Evaluation Studies as Topic</subj>
<subj type="unclass">Health Knowledge, Attitudes, Practice ethnology</subj>
<subj type="unclass">Health Services Accessibility organization & administration</subj>
<subj type="unclass">Humans</subj>
<subj type="unclass">Interviews as Topic</subj>
<subj type="unclass">Language</subj>
<subj type="unclass">Male</subj>
<subj type="unclass">Qualitative Research</subj>
<subj type="unclass">Social Networking</subj>
<subj type="unclass">Switzerland epidemiology</subj>
<subj type="unclass">Young Adult</subj>
</sug>
<ab>Eritreans comprise the largest group of asylum-seekers in Switzerland. Gaining recognized refugee status can take up to 36 months, during which time asylum-seekers live in a state of legal limbo, intensifying threats to their well-being. Resilience and mental health among this population is poorly understood. We interviewed 10 asylum-seekers residing in Switzerland using qualitative, in-depth interviews. Data were analyzed using the Framework Method. Results indicated that mental health was understood as a binary state rather than a continuum and that trusted friends and family were responsible for recognizing and attempting to treat mental health problems. Pathways to care were potentially interrupted for asylum-seekers. Capital building, considered through the lens of social resilience, consisted of language learning, establishing of new individual- and community-level social networks, and proactive symbolic capital building through volunteering. We contextualize the asylum-seekers' experience into a resilience framework and offer practical recommendations for improving mental health care access.</ab>
<pubtype>Journal Article</pubtype>
<doctype>Research Support, Non-U.S. Gov't</doctype>
</artinfo>
<language code="eng">English</language>
</controlInfo>
<displayInfo>
<pLink>
<url>https://search.ebscohost.com/login.aspx?
direct=true&AuthType=sso&db=mnh&AN=30222038&site=ehost-live&custid=s2775460</url>
</pLink>
</displayInfo>
</header>
</rec>
<rec resultID="16">
<header shortDbName="mnh" longDbName="MEDLINE with Full Text" uiTerm="29584520">
<controlInfo>
<bkinfo/>
<jinfo>
<jtl>The International journal of social psychiatry</jtl>
<jtl>Int J Soc Psychiatry</jtl>
<issn type="Electronic">1741-2854</issn>
</jinfo>
<pubinfo>
<dt year="2018" month="06" day="01">2018 Jun</dt>
<vid>64</vid>
<iid>4</iid>
<place>England</place>
</pubinfo>
<artinfo>
<ui type="pmid">29584520</ui>
<ui type="doi">10.1177/0020764018765237</ui>
<pages>359-366</pages>
<formats/>

<tig>
<atl>Coping, resilience and posttraumatic growth among Eritrean female refugees living in Norwegian asylum reception centres: A qualitative study.</atl>
</tig>
<aug>
<au>Abraham R</au>
<au>Lien L</au>
<au>Hanssen I</au>
</aug>
<sug>
<subj type="major">Adaptation, Psychological</subj>
<subj type="major">Refugees psychology</subj>
<subj type="major">Religion</subj>
<subj type="major">Social Support</subj>
<subj type="major">Transients and Migrants psychology</subj>
<subj type="unclass">Adolescent</subj>
<subj type="unclass">Adult</subj>
<subj type="unclass">Eritrea ethnology</subj>
<subj type="unclass">Female</subj>
<subj type="unclass">Focus Groups</subj>
<subj type="unclass">Humans</subj>
<subj type="unclass">Middle Aged</subj>
<subj type="unclass">Norway</subj>
<subj type="unclass">Qualitative Research</subj>
<subj type="unclass">Young Adult</subj>
</sug>
<ab>The links between migration and health are well documented. Being a refugee exacerbates migrants' vulnerability through trauma and loss. The aim of this study is to identify sources of resilience, coping and posttraumatic growth in female Eritrean refugees living in Norwegian asylum reception centres.</ab>
<ab>The study had a qualitative, descriptive and explorative design with two focus group interviews and 10 individual in-depth interviews. Participants included 18 female Eritrean refugees aged 18-60, who had obtained refugee status and were still living in an asylum reception centre. A content-focused hermeneutic analytic approach was used.</ab>
<ab>Interviewees described the challenges of pre-flight and flight trauma, conditions at the refugee centre, communication difficulties and the 'endless' waiting for transfer to a municipality. To cope, they found it helpful to focus on the future and to think positively. Fellowship with and support from fellow Eritrean refugees were essential as they became a proxy family and provided a strong ethnic identity. Their religious belief also helped them cope and gave them hope for the future.</ab>
<ab>The interviewees in this study perceived their psychological problems as a normal reaction to what they had been through. Religious belief was an important resilience factor, as was social support, especially from peers.</ab>
<ab>The interviewees' coping was based on the realization of their psychological reactions being normal while doing their utmost to focus on their aims and hopes for the future.</ab>
<pubtype>Journal Article</pubtype>
<doctype>Multicenter Study</doctype>
</artinfo>
<language code="eng">English</language>
</controlInfo>
<displayInfo>
<pLink>
<url>https://search.ebscohost.com/login.aspx?direct=true&AuthType=sso&db=mnh&AN=29584520&site=ehost-live&custid=s2775460</url>
</pLink>
</displayInfo>
</header>
</rec>
<rec resultID="17">
<header shortDbName="mnh" longDbName="MEDLINE with Full Text" uiTerm="26562101">
<controlInfo>
<bkinfo/>
<jinfo>
<jtl>American journal of public health</jtl>
<jtl>Am J Public Health</jtl>

<issn type="Electronic">1541-0048</issn>
</jinfo>
<pubinfo>
<dt year="2016" month="01" day="01">2016 Jan</dt>
<vid>106</vid>
<iid>1</iid>
<place>United States</place>
</pubinfo>
<artinfo>
<ui type="pmid">26562101</ui>
<ui type="doi">10.2105/AJPH.2015.302920</ui>
<pages>178-88</pages>
<formats>
<fmt type="PDF"/>
</formats>
<tig>
<atl>Risk Factors for and Behavioral Consequences of Direct Versus Indirect Exposure to Violence.</atl>
</tig>
<aug>
<au>Zimmerman GM</au>
<au>Posick C</au>
</aug>
<sug>
<subj type="geographic">Chicago</subj>
<subj type="major">Adolescent Behavior psychology</subj>
<subj type="major">Child Behavior Disorders psychology</subj>
<subj type="major">Crime Victims psychology</subj>
<subj type="major">Exposure to Violence psychology</subj>
<subj type="major">Social Behavior Disorders psychology</subj>
<subj type="unclass">Adolescent</subj>
<subj type="unclass">Adolescent Behavior ethnology</subj>
<subj type="unclass">Black or African American psychology</subj>
<subj type="unclass">Black or African American statistics & numerical data</subj>
<subj type="unclass">Chicago epidemiology</subj>
<subj type="unclass">Child</subj>
<subj type="unclass">Child Behavior Disorders etiology</subj>
<subj type="unclass">Cluster Analysis</subj>
<subj type="unclass">Exposure to Violence ethnology</subj>
<subj type="unclass">Female</subj>
<subj type="unclass">Hispanic or Latino psychology</subj>
<subj type="unclass">Hispanic or Latino statistics & numerical data</subj>
<subj type="unclass">Humans</subj>
<subj type="unclass">Male</subj>
<subj type="unclass">Minority Groups psychology</subj>
<subj type="unclass">Minority Groups statistics & numerical data</subj>
<subj type="unclass">Risk Factors</subj>
<subj type="unclass">Social Behavior Disorders ethnology</subj>
<subj type="unclass">Social Behavior Disorders etiology</subj>
<subj type="unclass">Urban Population</subj>
</sug>
<ab>Research suggests that direct exposure (personal victimization) and indirect exposure (witnessing or hearing about the victimization of a family member, friend, or neighbor) to violence are correlated. However, questions remain about the co-occurrence of these phenomena within individuals. We used data on 1915 youths (with an average age of 12 years at baseline) from the Project on Human Development in Chicago Neighborhoods to examine this issue. Results indicated that youths who tended to be personally victimized were also likely to witness violence; conversely, youths who disproportionately witnessed violence were relatively unlikely to experience personal victimization. In addition, direct and indirect exposures to violence were associated with subsequent adverse outcomes in similar ways. The key distinguishing factor was, rather, the cumulative level of violence (both direct and indirect) to which youths were exposed.</ab>
<pubtype>Journal Article</pubtype>
</artinfo>
<language code="eng">English</language>

```
      </controlInfo>
      <displayInfo>
        <pLink>
          <url>https://search.ebscohost.com/login.aspx?
          direct=true&AuthType=sso&db=mnh&AN=26562101&site=ehost-live&custid=s2775460</url>
        </pLink>
      </displayInfo>
    </header>
  </rec>
  <rec resultID="18">
    <header shortDbName="mnh" longDbName="MEDLINE with Full Text" uiTerm="23488506">
      <controlInfo>
        <bkinfo/>
        <jinfo>
          <jtl>American journal of public health</jtl>
          <jtl>Am J Public Health</jtl>
          <issn type="Electronic">1541-0048</issn>
        </jinfo>
        <pubinfo>
          <dt year="2013" month="05" day="01">2013 May</dt>
          <vid>103</vid>
          <iid>5</iid>
          <place>United States</place>
        </pubinfo>
        <artinfo>
          <ui type="pmid">23488506</ui>
          <ui type="doi">10.2105/AJPH.2012.301184</ui>
          <pages>861-7</pages>
          <formats>
            <fmt type="HTML Full Text"/>
            <fmt type="HTML Full Text with Images"/>
            <fmt type="PDF"/>
          </formats>
          <tig>
            <atl>Stigma, discrimination, or symptomatology differences in self-reported mental health
            between US-born and Somalia-born Black Americans.</atl>
          </tig>
          <aug>
            <au>Henning-Smith C</au>
            <au>Shippee TP</au>
            <au>McAlpine D</au>
            <au>Hardeman R</au>
            <au>Farah F</au>
          </aug>
          <sug>
            <subj type="geographic">Minnesota</subj>
            <subj type="geographic">Somalia</subj>
            <subj type="geographic">United States</subj>
            <subj type="major">Depressive Disorder ethnology</subj>
            <subj type="major">Mental Health ethnology</subj>
            <subj type="major">Mental Health Services statistics & numerical data</subj>
            <subj type="major">Social Stigma</subj>
            <subj type="unclass">Adolescent</subj>
            <subj type="unclass">Adult</subj>
            <subj type="unclass">Black or African American psychology</subj>
            <subj type="unclass">Aged</subj>
            <subj type="unclass">Aged, 80 and over</subj>
            <subj type="unclass">Attitude to Health ethnology</subj>
            <subj type="unclass">Black People psychology</subj>
            <subj type="unclass">Community-Based Participatory Research</subj>
            <subj type="unclass">Emigrants and Immigrants psychology</subj>
            <subj type="unclass">Emigrants and Immigrants statistics & numerical data</subj>
            <subj type="unclass">Female</subj>
            <subj type="unclass">Humans</subj>
            <subj type="unclass">Male</subj>
```

```
          <subj type="unclass">Middle Aged</subj>
          <subj type="unclass">Minnesota</subj>
          <subj type="unclass">Somalia ethnology</subj>
          <subj type="unclass">United States</subj>
          <subj type="unclass">White People psychology</subj>
          <subj type="unclass">Young Adult</subj>
        </sug>
        <ab>We examined differences in self-reported mental health (SRMH) between US-born and
        Somalia-born Black Americans compared with White Americans. We tested how SRMH was affected
        by stigma toward seeing a mental health provider, discrimination in the health care
        setting, or symptoms of depression.</ab>
        <ab>Data were from a 2008 survey of adults in Minnesota and were limited to US-born and
        Somalia-born Black and White Americans (n = 938).</ab>
        <ab>Somalia-born adults were more likely to report better SRMH than either US-born Black or
        White Americans. They also reported lower levels of discrimination (18.6%) than US-born
        Black Americans (33.4%), higher levels of stigma (23.6% vs 4.7%), and lower levels of
        depressive symptoms (9.1% vs 31.6%). Controlling for stigma, discrimination, and
        symptomatology, Somalia-born Black Americans reported better SRMH than White and Black
        Americans (odds ratio = 4.76).</ab>
        <ab>Mental health programming and health care providers who focus on Black Americans'
        mental health might be missing important sources of heterogeneity. It is essential to
        consider the role of race and ethnicity, but also of nativity, in mental health policy and
        programming.</ab>
        <pubtype>Journal Article</pubtype>
        <doctype>Comparative Study</doctype>
        <doctype>Research Support, Non-U.S. Gov't</doctype>
      </artinfo>
      <language code="eng">English</language>
    </controlInfo>
    <displayInfo>
      <pLink>
        <url>https://search.ebscohost.com/login.aspx?
        direct=true&AuthType=sso&db=mnh&AN=23488506&site=ehost-live&custid=s2775460</url>
      </pLink>
    </displayInfo>
  </header>
</rec>
<rec resultID="19">
  <header shortDbName="mnh" longDbName="MEDLINE with Full Text" uiTerm="29621896">
    <controlInfo>
      <bkinfo/>
      <jinfo>
        <jtl>Anxiety, stress, and coping</jtl>
        <jtl>Anxiety Stress Coping</jtl>
        <issn type="Electronic">1477-2205</issn>
      </jinfo>
      <pubinfo>
        <dt year="2018" month="07" day="01">2018 Jul</dt>
        <vid>31</vid>
        <iid>4</iid>
        <place>England</place>
      </pubinfo>
      <artinfo>
        <ui type="pmid">29621896</ui>
        <ui type="doi">10.1080/10615806.2018.1460662</ui>
        <pages>459-474</pages>
        <formats/>
        <tig>
          <atl>Stigma modifies the association between social support and mental health among
          sexual violence survivors in the Democratic Republic of Congo: implications for practice.
          </atl>
        </tig>
        <aug>
          <au>Wachter K</au>
          <au>Murray SM</au>
```

```
          <au>Hall BJ</au>
          <au>Annan J</au>
          <au>Bolton P</au>
          <au>Bass J</au>
        </aug>
        <sug>
          <subj type="geographic">Democratic Republic of the Congo</subj>
          <subj type="major">Anxiety Disorders psychology</subj>
          <subj type="major">Depressive Disorder psychology</subj>
          <subj type="major">Sex Offenses psychology</subj>
          <subj type="major">Social Stigma</subj>
          <subj type="major">Social Support</subj>
          <subj type="major">Stress Disorders, Post-Traumatic psychology</subj>
          <subj type="major">Survivors psychology</subj>
          <subj type="unclass">Adult</subj>
          <subj type="unclass">Anxiety Disorders diagnosis</subj>
          <subj type="unclass">Anxiety Disorders therapy</subj>
          <subj type="unclass">Correlation of Data</subj>
          <subj type="unclass">Cross-Sectional Studies</subj>
          <subj type="unclass">Democratic Republic of the Congo</subj>
          <subj type="unclass">Depressive Disorder diagnosis</subj>
          <subj type="unclass">Depressive Disorder therapy</subj>
          <subj type="unclass">Developing Countries</subj>
          <subj type="unclass">Female</subj>
          <subj type="unclass">Humans</subj>
          <subj type="unclass">Internal-External Control</subj>
          <subj type="unclass">Mental Health</subj>
          <subj type="unclass">Middle Aged</subj>
          <subj type="unclass">Psychotherapy, Group</subj>
          <subj type="unclass">Socioeconomic Factors</subj>
          <subj type="unclass">Stress Disorders, Post-Traumatic diagnosis</subj>
          <subj type="unclass">Stress Disorders, Post-Traumatic therapy</subj>
          <subj type="unclass">Young Adult</subj>
        </sug>
        <ab>The aim of this study was to further understanding of the relationship between social
        support, internalized and perceived stigma, and mental health among women who experienced
        sexual violence in the eastern Democratic Republic of Congo (DRC).</ab>
        <ab>Drawing from baseline survey data collected in eastern DRC, researchers conducted a
        secondary cross-sectional analysis using data from 744 participants. Regression and
        moderation analyses were conducted to examine associations between social support
        variables, felt stigma, and depression, anxiety and posttraumatic stress disorder (PTSD).
        </ab>
        <ab>Emotional support seeking and felt stigma were positively associated with increased
        symptom severity across all three mental health variables. Stigma modified associations
        between emotional support seeking and depression (t = -2.49, p = .013), anxiety (t = -3.08,
        p = .002), and PTSD (t = -2.94, p = .003). Increased frequency of emotional support seeking
        was associated with higher mental health symptoms of anxiety and PTSD among women
        experiencing all levels of stigma.</ab>
        <ab>Enhancing understanding of social support and stigma may inform research and
        intervention among Congolese forced migrant populations across circumstances and geographic
        locations. Implications for practice and research are discussed.</ab>
        <pubtype>Journal Article</pubtype>
        <doctype>Comparative Study</doctype>
        <doctype>Randomized Controlled Trial</doctype>
      </artinfo>
      <language code="eng">English</language>
    </controlInfo>
    <displayInfo>
      <pLink>
        <url>https://search.ebscohost.com/login.aspx?
        direct=true&AuthType=sso&db=mnh&AN=29621896&site=ehost-live&custid=s2775460</url>
      </pLink>
    </displayInfo>
  </header>
</rec>
```

<rec resultID="20">
<header shortDbName="mnh" longDbName="MEDLINE with Full Text" uiTerm="23391152">
<controlInfo>
<bkinfo/>
<jinfo>
<jtl>AIDS care</jtl>
<jtl>AIDS Care</jtl>
<issn type="Electronic">1360-0451</issn>
</jinfo>
<pubinfo>
<dt year="2013" month="01" day="01">2013</dt>
<vid>25</vid>
<iid>9</iid>
<place>England</place>
</pubinfo>
<artinfo>
<ui type="pmid">23391152</ui>
<ui type="doi">10.1080/09540121.2013.763892</ui>
<pages>1193-8</pages>
<formats>
<fmt type="PDF"/>
</formats>
<tig>
<atl>Toward the integration of comprehensive mental health services in HIV care: an assessment of psychiatric morbidity among HIV-positive individuals in sub-Saharan Africa.
</atl>
</tig>
<aug>
<au>Olagunju AT</au>
<au>Ogundipe OA</au>
<au>Erinfolami AR</au>
<au>Akinbode AA</au>
<au>Adeyemi JD</au>
</aug>
<sug>
<subj type="geographic">Africa South of the Sahara</subj>
<subj type="major">HIV Infections psychology</subj>
<subj type="major">Mental Disorders therapy</subj>
<subj type="major">Mental Health Services organization & administration</subj>
<subj type="unclass">Adult</subj>
<subj type="unclass">Africa South of the Sahara</subj>
<subj type="unclass">Female</subj>
<subj type="unclass">Humans</subj>
<subj type="unclass">Male</subj>
<subj type="unclass">Middle Aged</subj>
<subj type="unclass">Morbidity</subj>
<subj type="unclass">Prevalence</subj>
<subj type="unclass">Sampling Studies</subj>
<subj type="unclass">Surveys and Questionnaires</subj>
</sug>
<ab>Existing evidence from research supports the desirability of integration of mental health services into HIV care in order to mitigate the grave consequences of unattended mental health morbidity among People Living with HIV/AIDS (PLWHA). This study aims to find out the burden and pattern of psychiatric disorders that is prevalent among HIV-positive individuals attending a Nigerian-based HIV clinic. The study participants, consisting of 295 HIV-positive adults were recruited using systematic random sampling method. The participants were subjected to questionnaire to elicit demographic profile and General Health Questionnaire (GHQ-28) to screen for probable psychiatric disorders. This was followed by Structured Clinical Interview for DSM-IV Non patient (SCID-NP) to diagnose the presence of psychiatric morbidity in any of the participant with a GHQ-28 score ≥5 and 10% of those with GHQ-28 score <5. Data were analyzed with SPSS 17. Of the 295 participants interviewed, approximately one-quarter (25.1%) of the participants had diagnosable psychiatric illness based on SCID-NP. Depression was the commonest mental disorder detected as 44 (14.9%) met the DSM-IV Axis 1 diagnosis of major depressive disorders. Anxiety disorders, concurrent Nicotine with Alcohol dependence and cannabis abuse were elicited in

24 (8.1%), 4 (1.3%), and 2 (0.7%) participants, respectively. This study finds a higher burden of psychiatric disorders in PLWHA in comparison to what is obtainable in the general population based on previous research works in similar context. Thus further underscores the need for integration of comprehensive psychiatric services into HIV care. We advocate the support and commitment of key stakeholders in HIV care to the translation of this research-based evidence into practice among PLWHA.</ab>
<pubtype>Journal Article</pubtype>
</artinfo>
<language code="eng">English</language>
</controlInfo>
<displayInfo>
<pLink>
<url>https://search.ebscohost.com/login.aspx?direct=true&AuthType=sso&db=mnh&AN=23391152&site=ehost-live&custid=s2775460</url>
</pLink>
</displayInfo>
</header>
</rec>
<rec resultID="21">
<header shortDbName="mnh" longDbName="MEDLINE with Full Text" uiTerm="28766372">
<controlInfo>
<bkinfo/>
<jinfo>
<jtl>Anxiety, stress, and coping</jtl>
<jtl>Anxiety Stress Coping</jtl>
<issn type="Electronic">1477-2205</issn>
</jinfo>
<pubinfo>
<dt year="2017" month="11" day="01">2017 Nov</dt>
<vid>30</vid>
<iid>6</iid>
<place>England</place>
</pubinfo>
<artinfo>
<ui type="pmid">28766372</ui>
<ui type="doi">10.1080/10615806.2017.1361936</ui>
<pages>702-715</pages>
<formats/>
<tig>
<atl>Role of event centrality and emotion regulation in posttraumatic stress disorder symptoms among internally displaced persons.</atl>
</tig>
<aug>
<au>Chukwuorji JC</au>
<au>Ifeagwazi CM</au>
<au>Eze JE</au>
</aug>
<sug>
<subj type="major">Adaptation, Psychological</subj>
<subj type="major">Emotions</subj>
<subj type="major">Stress Disorders, Post-Traumatic psychology</subj>
<subj type="major">Transients and Migrants psychology</subj>
<subj type="unclass">Adolescent</subj>
<subj type="unclass">Adult</subj>
<subj type="unclass">Aged</subj>
<subj type="unclass">Aged, 80 and over</subj>
<subj type="unclass">Child</subj>
<subj type="unclass">Cross-Sectional Studies</subj>
<subj type="unclass">Female</subj>
<subj type="unclass">Humans</subj>
<subj type="unclass">Male</subj>
<subj type="unclass">Middle Aged</subj>
<subj type="unclass">Nigeria</subj>
<subj type="unclass">Surveys and Questionnaires</subj>
<subj type="unclass">Young Adult</subj>
