# Supplementary material for: Mental health and mental health help-seeking behaviors among first-generation voluntary African migrants: A systematic review
Source: PLoS One. 2024 Mar 18;19(3):e0298634. doi: 10.1371/journal.pone.0298634 (PMC10947684; doi:10.1371/journal.pone.0298634)
Supplement: Attachment — Submitted filename: Responses to Reviewers.docx [file pone.0298634.s006.docx]

03/01.2023

We thank the Editor and reviewers for the time and insightful comments made on this work, and have addressed each below. Changes have also been made in relevant sections of the manuscript and other related documents.

| **Paper section** | **Reviewer comments** | **Author’s response** |
| --- | --- | --- |
| **Journal requiremrnts** | 1. Please ensure that your manuscript meets PLOS ONE's style requirements, including those for file naming. The PLOS ONE style templates can be found at  [https://journals.plos.org/plosone/s/file?id=wjVg/PLOSOne_formatting_sample_main_body.pdf](https://protect-au.mimecast.com/s/B552CK1D1wsMLXA5SoznlQ?domain=journals.plos.org) and  [https://journals.plos.org/plosone/s/file?id=ba62/PLOSOne_formatting_sample_title_authors_affiliations.pdf](https://protect-au.mimecast.com/s/dmWpCL7E7xcwLGonUKmjHo?domain=journals.plos.org) | We thank the reviewer for this note, and have reviewed all sections of the submission to ensure adherence to the PLOS ONE style. |
|  | 2. We note that the grant information you provided in the ‘Funding Information’ and ‘Financial Disclosure’ sections do not match.   When you resubmit, please ensure that you provide the correct grant numbers for the awards you received for your study in the ‘Funding Information’ section. | This has been reviewed. |
|  | 3. We note that you have indicated that data from this study are available upon request. PLOS only allows data to be available upon request if there are legal or ethical restrictions on sharing data publicly. For more information on unacceptable data access restrictions, please see [awqeqweasdas://journals.plos.org/plosone/s/data-availability#loc-unacceptable-data-access-restrictions](https://protect-au.mimecast.com/s/lC4mCMwGwySkjwo4COV8xE?domain=journals.plos.org).   In your revised cover letter, please address the following prompts:  a) If there are ethical or legal restrictions on sharing a de-identified data set, please explain them in detail (e.g., data contain potentially sensitive information, data are owned by a third-party organization, etc.) and who has imposed them (e.g., an ethics committee). Please also provide contact information for a data access committee, ethics committee, or other institutional body to which data requests may be sent.  b) If there are no restrictions, please upload the minimal anonymized data set necessary to replicate your study findings as either Supporting Information files or to a stable, public repository and provide us with the relevant URLs, DOIs, or accession numbers. For a list of acceptable repositories, please see [http://journals.plos.org/plosone/s/data-availability#loc-recommended-repositories](https://protect-au.mimecast.com/s/bGVICNLJLzFPoOnmu8XBs3?domain=journals.plos.org).  We will update your Data Availability statement on your behalf to reflect the information you provide. | We thank the reviewers for this observation and would like to note that there are no restrictions on the data associated with this review. We have hence made the data available by uploading the minimal anonymized dataset necessary to replicate this study as supporting information:  **S1 File**  **S1A Appendix to S3D Appendix** |
|  | 4. Please note that in order to use the direct billing option the corresponding author must be affiliated with the chosen institute. Please either amend your manuscript to change the affiliation or corresponding author, or email us at plosone@plos.org with a request to remove this option. | Thanks. Dr Levi Uchechukwu Osuagwu is a co-corresponding author on this work, and is affiliated to the nominated institution – Western Sydney University. |
|  | 5. We are unable to open your Supporting Information file CINAHL Complete_15.07.2022.2022. Please kindly revise as necessary and re-upload | This PDF file has been uploaded again for your perusal. It contains the CINAHL Complete search strategy, and has been renamed as:  **S1A Appendix.** |
|  | 6. Please include captions for your Supporting Information files at the end of your manuscript, and update any in-text citations to match accordingly. Please see our Supporting Information guidelines for more information: [http://journals.plos.org/plosone/s/supporting-information](https://protect-au.mimecast.com/s/mk5uCOMKMASZ37PmIYnuBx?domain=journals.plos.org). | This information has been included at the end of the manuscript as follows:  Supporting Information Caption  **S1A Appendix.** CINAHL Search Strategy 15.07.2022  **S1B Appendix.** Embase Search Strategy 15.07.2022  **S1C Appendix.** Medline Complete Search Strategy 15.07.2022  **S1D Appendix.** PsychInfo Search Strategy 15.07.2022  **S2A Appendix.** CINAHL Search Strategy 23.05.2023  **S2B Appendix.** Embase Search Strategy 23.05.2023  **S2C Appendix.** Medline Complete Search Strategy 23.05.2023  **S2D Appendix.** PsychInfo Search Strategy 23.05.2023  **S3A Appendix.** CINAHL Search Strategy 16.12.2023  **S3B Appendix.** Embase Search Strategy 17.12.2023  **S3C Appendix.** Medline Complete Search Strategy 16.12.2023  **S3D Appendix.** PsychInfo Search Strategy 17.12.2023 |
|  | Thank you so much for submitting your manuscript to PLOS One. It was a pleasure reading and learning about your work and finding scholarly merit and practical relevance in this work. However, I believe it will benefit from improved quality and readability taking into consideration the following comments/concerns before accepting for publication in this journal. Here are the concerns to improve the manuscript: | We thank the reviewer for their time and kind comments. |
|  | Abstract section para 44 either changes the objective to an introduction or makes a very clear objective and states only the objective not background information. | We appreciate this comment and have addressed it by deleting the subheadings in the abstract section. |
|  | Methods section add a sentence about how you synthesised data. | A summary of the data synthesis process has been included in the methods section of the abstract |
|  | • Para 91 -92 reference number 14 add one more citation or correct the writing partners of para 91. | In response to this comment and the next three comments, we have reviewed paragraph 3 of the introduction.  We have added an additional reference:  **Lines 89 to 94** |
|  | Similar way para 93 studies have …. But only one reference is evident in para 96 ref number 96. Either add references or change the way of presentation of para 93-96. | The line about mental health risk in African men has been rephrased to address this comment. See changes in:  **Lines 90 to 94**  To Address the query in sentence in question has been updated, an additional reference has been added to back the statement. See changes in: **Lines 90 to 94** |
|  | Insert a citation in para 102-112 to claim your statement. | References have been added to address this concern:  **Lines 104 to 117** |
|  | • Citations are missing in para 115 – 123 to claim your arguments. | We thank the reviewer for this observation. References have been added to applicable sections of paragraph. See changes in:  **Lines** **118 to 164** |
|  | • Make consistency of your writing in headings and sub-headings first letter is capitalised and small i.e. 2.1 and 2.2 which are not consistent. | Good point. All subheadings have been reviewed to ensure that sentence case is used.  **See changes in whole manuscript.** |
|  | • Make a consistent presentation and correct para 142-143 and state January 2012 – May 2023. | This sentence has been corrected. See changes in:  **Lines** **147** |
|  | • Make consistency para 146 including your data including month in 2012 which is missing and explain the reason why only chose 2012 which is not clear. | The missing month January has been added to 2012.  **Line 147**  A rational for limiting this search to 2012 has also been provided.  See changes in:  **Methods, Inclusion Criteria #6** |
|  | • Methods section there is no data synthesis section and not clear how you synthesise your data in this review which should be clearly stated in detail under the methods section and a key sentence under the abstract methods section as well. | We thank the reviewer for this observation. Approaches used for data synthesis have been mentioned. See changes in:   - **Methods section of the abstract** - **Methods, under Data extraction and synthesis** |
|  | • Define the first-time used abbreviations i.e. STROBE, PRISMA, and follow the entire manuscript. | The following has been done in response to this request:  STROBE – has been written in full. The acronym is not provided since this term is not mentioned again in the article.  PRISMA is already defined in the article. See first paragraph of methods. |
|  | • Table 1 research design needs to correct the study design section where qualitative and, Feminist studies are stated in the research design. So consult with the research methods book and state the correct research design of each paper and state it consistently in the entire manuscript. | In response to this comment, the reference to Feminist anthropology has been replace with qualitative study, highlighting the qualitative approach used. See changes in:   - **See methods of Baiden & Evans study in Table 1.**   Design used in all included studies have also been provided. |
|  | • Table 2 Check the paper – Orjiako et al 2013 in para 227 and your reference list which is not consistent in the year of the publication. so check all references and strongly suggest using reference management software to make consistency on the citation and reference lists. | Mendeley was used to reference all papers presented in this manuscript. As the reviewer may be aware, sometimes the initial printed PDF versions of papers do not always have the final citation details since it takes time for these to be finalised. Hence, inconsistencies may occur between the date on the PDF version, and date on updated databases like Google Scholar and Mendeley. This is exactly the situation for this paper. The PDF version we extracted has 2013 on it, but Mendeley had the updated data (2014), hence the omission. We appreciate the reviewer calling our attention to this situation and have addressed it in all relevant sections of the paper. |
|  | • Table 2 Orjiako et al 2013 paper, extracts the consistent data i.e. 95%CI which is missing. | We would like to call the reviewer’s attention to the fact that no confidence intervals are presented in Orijiako & So’s paper (See Table 2 of paper). As we have accurately presented, the *p*-values reported in the paper are sufficient for the purposes of this review. |
|  | • Table 2 Insert repeat header rows which we can see section heading on each page. | We appreciate this suggestion and have applied this change to Tables 1 and 2. |
|  | • Para 277 3.6 remove (RoB) but you can define in para 279. | We have removed RoB from the heading as recommended. See changes in:   - **Results, Risk of Bias heading**   Please note that RoB has already been defined in the methods section, so this definition as not been repeated in the results section. |
|  | • The discussion section of this manuscript should be very clear, concise and coherent using both empirical and policy sources but para 296 to 316, 320-329 and 341-356 have been written without a single citation/reference. You have not used any single reference. | In response to this comment, we have not included any new references in paragraph one since gold standard academic writing suggests that the first paragraph is used to reiterate the study aims and to summarize of the findings, which is what we have done. **See Discussion, Paragraph 1**.  We have however done a thorough review of the rest of the discussion to ensure it is concise and included more references, where necessary. **See changes discussion.** |
|  | • Para 4.1 adds strengths in this section, and Para 365 and 377 sections 4.2 & 4.3 should merge under the conclusion section. Recommendations should come after the conclusion of your writing. | We thank the reviewer for these suggestions, and have made all suggested changed:  **See changes discussion and conclusion.** |
|  | • Section 5 conclusion para 383-385 methods stuff should not be repeated in the conclusion section but draw your conclusion and also add present the recommendation section. | This statement has been amended per the reviewer’s recommendation:  **See conclusion.** |
|  | • PRASMA flow chart states only those points which have a number and removes those which do not have a number i.e. only (n=0) section | We have duly updated the PRISMA flow diagram per this recommendation:  **See Fig 1. PRISMA Flow Diagram** |
| **Reviewer 1** | | |
| General comments | 1. The manuscript is technically sound. However, based on the details of the review, the conclusion part is less reflective of what particularly has been identified. | We thank the reviewer for these kind comments and have amended the conclusion per this recommendation.  **See conclusion.** |
|  | 2. It is already mentioned in the study limitation that the number of studies included in the review is very small, it would have been more insightful if there were more studies included in the review. | In response to this and Reviewer 2s comments, we have repeated the search and added papers (4) that meet the study criteria.  See changes in all sections of the manuscript. |
|  | 3. Eventhough the study follows PRISMA flowchart for selection of the studies and has been shown clearly, the selection of studies and its relevence to the study purpose is of concern. As the title of the study focuses on mental health and help seeking among african migrants, selection of women with post natal depression can possibly reflect mental health conditions and help seeking behavior because of specific factors related to child birth rather than just reflecting the concern as a migrant. | We thank the reviewer for this comment. We would like to mention that this review focused on mental health and help-seeking behavior in first-generation voluntary African migrants, and not just in African migrants.  We included studies that focused on postnatal depression in women meeting our study criteria, specifically first-generation African migrants. Even though postnatal depression is linked to childbirth, it is a mental health concern in the targeted migrant group, highlighting challenges faced by African women having children away from their home country.  To address this, we have separated the results from studies on postnatal depression from the results on other mental health challenges. See changes in:  **Results**  **Discussion** |
|  | 4. The reviewer find the generalizability issue of this study. Very small number of articles has been included with lots of limitations. Authors should make it clear what they are trying to conclude /extract from this study? | We allude to the reviewer’s concerns and have amended the conclusions accordingly.  **See conclusion.** |
| **Reviewer 2** | | |
|  | Thank you for the opportunity to review this systematic review. The review starts off strong with a well written introduction and study objectives. However, the search strategy that the authors have used to find peer-reviewed articles from Africa includes generic geographic search terms (e.g. Africa or Sub-Saharan Africa), but does not include specific African countries (e.g. Ghana, Nigeria). This has potentially led to exclusion of several eligible studies. Secondly, while the four studies the authors have included provide preliminary insights on a few African countries, and a few migrant sub-categories, the limited no. of studies do not provide sufficient findings to answer the research questions. Kindly refer to the following detailed comments for each section: | We thank the reviewer for their kind words about how this review started off.  In response to the comment about the search terms used, we have repeated the search, including a list of all African countries. |
| Abstract | The review objective is clearly stated. | Thank you. |
|  | Line 58: Typo in "Results" subsection "two quantitative and two quantitative studies". I suppose the authors meant two quantitative and two qualitative studies. | We thank the reviewer for noting this mistake and have made the necessary change.  **See Abstract, Results section** |
|  | Line 60-61: "Risk and protective factors associated with mental health and the barriers and facilitators of mental health help-seeking behaviour were identified." - What were these risk & protective factors, and barriers & facilitators? Mention briefly. | We appreciate this suggestion, and have provided examples for each of these listed variables:  **See Abstract, Results section** |
|  | Line 64: Conclusion presented in the abstract not justified by results presented in abstract. | The conclusion has been amended in response to this comment, with efforts made to just focus on the study findings.  **See Abstract, Conclusion section** |
| Introduction | This section follows a logical and clear argument, is informative and supported by good references. Rationale for selecting the population of interest is well-justified. The research questions are also clear. | We thank the reviewer for these kind comments. |
|  | Line 73: More recent data on no. of migrants available from IOM World Migration Report 2022/23. | We thank the reviewer for sharing this information. |
|  | Line 98: Explain the term "affirming practices" | A brief explanation to this term has been provided:  **See Introduction, Paragraph 3** |
|  | Could the authors clarify or add a sentence on whether the population of interest includes students, labour migrants, migrants moving upon marriage, etc.? | We appreciate this suggestion. These have been specified in:   - **Introduction, Paragraph 4** - **Methods, Under Inclusion and Exclusion criteria.** |
| Methods and materials | Methods section is well-written with good description, particularly of the quality appraisal or risk of bias section. | We thank the reviewer for this kind observation. |
|  | Line 137-138: Not clear why this line says African migrants to Western countries. The inclusion criteria does not indicate migration to Western countries only. | We thank the reviewer for this observation. Our review focuses on African migrants outside the continent of Africa, so not necessarily African migrants in Western countries. This has been specified in:   - **Abstract** - **Introduction, last paragraph** - **Methods, Inclusion criteria 4** |
|  | Line 142: Provide a rationale for selecting the time frame 2012-2023 Search strategy in supplementary file: From the search strategy, I can see that the authors have used "Africa/African" as their search term. This might have led to exclusion of studies that mentioned specific countries such as Nigeria or Ghana rather than Africa. Also including the name of key destination countries could have yielded more results. For example, by using "Sudan" and "UAE" (destination country) in your search strategy, I found: Frontiers \| Psychological Distress and Homesickness Among Sudanese Migrants in the United Arab Emirates (frontiersin.org). And by using "Ghana" and "UK" in the search strategy, I found: Perceptions of Ghanaian Migrant Mothers Living in London towards Postnatal Depression during Postnatal Periods There may be many such missed papers. Could the authors please justify this? This is important because the review has included only 4 papers. | - We have provided a rationale for the time frame specified. See:   **Methods, Inclusion Criteria #6**   - In response to the comment about the search terms used, and the resultant limited number of papers included, we have repeated the search, including a list of all African countries. Four additional papers have been added to this review that met the search criteria. |
| Results | Line 276 Suggest replacing the term "non-postnatal depression studies" with the "quantitative study by Orjiako". The four studies included are quite different from one another in terms of methodology and also the population of interest, because of which there isn't enough data to synthesize or find patterns meaningfully. Similarly, it is not clear which African countries have been included in all the four studies, and therefore it is not easy to ascertain to what extent the various countries in Africa have been represented. African nations are diverse in their culture, economy and socio-political situation. Therefore, the four studies are not adequate in representing the mental health impact on migrants from Africa. | Reference to ‘non-postnatal depression studies’ has been deleted in this section of the results. See change in   - **Results, Mental health and help-seeking behaviours, Paragraph 1**   We do acknowledge the reviewer’s comment about the distinct methodological approaches used across the included studies and how that limits the quality of data synthesis. We have tried to specify the methods used for each study in the results, reported and acknowledged in the study limitations. See change in:   - **Results** - **Discussion, Review strengths and limitations, Limitation # 2**   We agree with the reviewer that the findings of this review has limited generalisability due to the limited number of countries represented, and the lack of information on the country of origin of some samples. The countries represented across studies has been included in the results and this has been included as a study limitation.  See change in:   - **Results, Study characteristics** - **Discussion, Review strengths and limitations, Limitation # 4** |
|  | Most importantly, the findings from each study have not been linked to key contextual aspects such as the population, destination country, country of origin and other contextual factors among others. The authors have stated the findings but have not discussed how it links to the migrants culture in their home country, or the culture/circumstances in the destination country. | We appreciate the reviewer’s comment and have revised the discussion to address the comment. Although this is hard to do for each finding in the context of a systematic review, we have done that for a few finding (e.g., discussion about English proficiency and Type A trait). See changes in:   - **Discussion** |
|  | Because of limited studies, many migrant sub-categories are not well represented including student groups/labour migrants, married vs. unmarried migrants, younger vs. older migrants, etc. While the authors have presented gender disaggregated sample, they have not discussed whether or how mental health impacts/help-seeking vary among men and women. | We note the comment about the limited generalizability of the results in relation to the migrant subgroups represented, and have included this as a study limitation:   - **Discussion, Review strengths and Limitations, Limitation #5**   While the topic of gender differences is interesting, that was not the focus of this review. We would also like to mention to the reviewer that the papers included in this review did not test gender differences, so there are no evidence-based findings to discuss on that topic in the context of this review. Thanks. |
| Discussion | Line 303 "…fear of mental health stigma within certain cultural backgrounds."- What kind of cultural backgrounds? These need to be discussed. | This statement has been deleted in the current version of the discussion as part of the revisions. |
|  | Line 338 "relational factors"- It is not clear which relationships does relational factors include. The authors have specified health professionals, but what about partners, neighbours, colleagues, other family members, etc. Are they included in "relational factors"? | We thank the reviewer for this note. This phrase has been deleted in the current version of the discussion as part of the revisions. Please note that the revised version of the discussion has been updated to include the findings from the added studies:   - **Discussion** |
